# GDF15 linked to maternal risk of nausea and vomiting during pregnancy

**DOI:** 10.1038/s41586-023-06921-9

**Published:** 2023-12-13

**Authors:** M. Fejzo, N. Rocha, I. Cimino, S. M. Lockhart, C. J. Petry, R. G. Kay, K. Burling, P. Barker, A. L. George, N. Yasara, A. Premawardhena, S. Gong, E. Cook, D. Rimmington, K. Rainbow, D. J. Withers, V. Cortessis, P. M. Mullin, K. W. MacGibbon, E. Jin, A. Kam, A. Campbell, O. Polasek, G. Tzoneva, F. M. Gribble, G. S. H. Yeo, B. Y. H. Lam, V. Saudek, I. A. Hughes, K. K. Ong, J. R. B. Perry, A. Sutton Cole, M. Baumgarten, P. Welsh, N. Sattar, G. C. S. Smith, D. S. Charnock-Jones, A. P. Coll, C. L. Meek, S. Mettananda, C. Hayward, N. Mancuso, S. O’Rahilly

**Affiliations:** 1 Center for Genetic Epidemiology, Department of Population and Public Health Sciences, Keck School of Medicine, University of Southern California, Los Angeles, CA USA; 2 Medical Research Council (MRC) Metabolic Diseases Unit, Institute of Metabolic Science, University of Cambridge, Cambridge, UK; 3 Peptidomics and Proteomics Core Facility, Institute of Metabolic Science, University of Cambridge, Cambridge, UK; 4 Core Biochemical Assay Laboratory, Cambridge University Hospitals NHS Foundation Trust, Cambridge, UK; 5 Department of Paediatrics, Faculty of Medicine, University of Kelaniya, Thalagolla Road, Ragama, Sri Lanka; 6 Adolescent and Adult Thalassaemia Care Center (University Medical Unit), North Colombo Teaching Hospital, Kadawatha, Sri Lanka; 7 Department of Medicine, Faculty of Medicine, University of Kelaniya, Ragama, Sri Lanka; 8 Department of Obstetrics and Gynaecology, University of Cambridge, NIHR Cambridge Biomedical Research Centre, Cambridge, UK; 9 Centre for Trophoblast Research (CTR), Department of Physiology, Development and Neuroscience, University of Cambridge, Cambridge, UK; 10 Department of Population and Public Health Sciences, Keck School of Medicine, University of Southern California, Los Angeles, CA USA; 11 Department of Obstetrics and Gynaecology, Keck School of Medicine, University of Southern California, Los Angeles, CA USA; 12 Hyperemesis Education and Research Foundation, Clackamas, OR USA; 13 Centre for Genomic and Experimental Medicine, Institute of Genetics and Cancer, University of Edinburgh, Edinburgh, UK; 14 Faculty of Medicine, University of Split, Split, Croatia; 15 Regeneron Genetics Center, Tarrytown, NY USA; 16 Department of Paediatrics, University of Cambridge, Cambridge, UK; 17 MRC Epidemiology Unit, Institute of Metabolic Science, University of Cambridge, Cambridge, UK; 18 Department of Obstetrics and Gynaecology, Cambridge University Hospitals NHS Foundation Trust, Cambridge, UK; 19 School of Cardiovascular and Metabolic Health, University of Glasgow, Glasgow, UK; 20 University Paediatrics Unit, Colombo North Teaching Hospital, Ragama, Sri Lanka; 21 MRC Human Genetics Unit, Institute of Genetics and Cancer, University of Edinburgh, Edinburgh, UK; 22 Department of Quantitative and Computational Biology, University of Southern California, California, CA USA; 23 Norris Comprehensive Cancer Center, Keck School of Medicine, University of Southern California, California, CA USA; 24 NIHR Cambridge Biomedical Research Centre, Cambridge, UK

**Keywords:** Translational research, Reproductive disorders, Genetic association study

## Abstract

GDF15, a hormone acting on the brainstem, has been implicated in the nausea and vomiting of pregnancy, including its most severe form, hyperemesis gravidarum (HG), but a full mechanistic understanding is lacking^1–4^. Here we report that fetal production of GDF15 and maternal sensitivity to it both contribute substantially to the risk of HG. We confirmed that higher GDF15 levels in maternal blood are associated with vomiting in pregnancy and HG. Using mass spectrometry to detect a naturally labelled GDF15 variant, we demonstrate that the vast majority of GDF15 in the maternal plasma is derived from the feto-placental unit. By studying carriers of rare and common genetic variants, we found that low levels of GDF15 in the non-pregnant state increase the risk of developing HG. Conversely, women with β-thalassaemia, a condition in which GDF15 levels are chronically high^5^, report very low levels of nausea and vomiting of pregnancy. In mice, the acute food intake response to a bolus of GDF15 is influenced bi-directionally by prior levels of circulating GDF15 in a manner suggesting that this system is susceptible to desensitization. Our findings support a putative causal role for fetally derived GDF15 in the nausea and vomiting of human pregnancy, with maternal sensitivity, at least partly determined by prepregnancy exposure to the hormone, being a major influence on its severity. They also suggest mechanism-based approaches to the treatment and prevention of HG.

## Abstract (web summary)

Elevated circulating levels of GDF15 in pregnant women are associated with severe nausea and vomiting, and sensitivity to such symptoms during pregnancy is partly determined by prepregnancy levels of this hormone.

## Main

Nausea and vomiting affect approximately 70% of human pregnancies and are often debilitating^2^. Hyperemesis gravidarum (HG) is diagnosed when nausea and vomiting are so severe that women are unable to eat and/or drink normally and have greatly limited daily activity. This is frequently accompanied by weight loss and electrolyte disturbance, which can carry significant risks to the longer-term health of both mother and offspring^2^. In the USA, HG is the leading cause of hospitalization in early pregnancy and the second most common cause of pregnancy hospitalization overall^6^. Until recently there has been no significant advance in the understanding of the molecular pathogenesis of nausea and vomiting of pregnancy (NVP) or HG. A body of evidence implicating GDF15, a circulating member of the TGFß superfamily, in these disorders has been emerging. In the non-pregnant state, GDF15 is ubiquitously produced in response to a range of cellular stresses. Its receptor, a heterodimer of GFRAL and RET, is expressed only in the hindbrain where its activation leads to nausea, vomiting and aversive responses^7,8^. For example, *cis*-platinum chemotherapy acutely elevates circulating GDF15 and the vomiting that occurs as a result of this is, in non-human primates, largely prevented by neutralizing GDF15 (ref. ^9^). The presence of high levels of GDF15 (then called MIC-1) in maternal blood in normal human pregnancy was first reported in 2000 (ref. ^10^) by Breit and colleagues who first described the hormone. Recently, GDF15 was found to be one of the most abundant peptides secreted from human trophoblast organoids^11^ and *GDF15* mRNA is more abundant in placental mRNA than in all other tissues examined by the GTEx consortium^12^. When compared to women who had low levels of nausea or vomiting, concentrations of GDF15 in maternal circulation have been reported to be higher in women experiencing vomiting in pregnancy^13^ and in a small group of women with HG^14^. These findings need to be viewed in the light of subsequent evidence for biased detection of common isoforms of GDF15 by the assays used^15^. The notion that GDF15 may have a primary role in the aetiology of HG, rather than increase as a consequence of the condition, was supported by the findings of the first genome-wide association study of women with HG, which reported several independent variants close to the *GDF15* gene as the most highly associated single nucleotide polymorphism (SNPs) in the maternal genome^3^. Subsequently, Fejzo et al. undertook an exome sequencing study in HG cases and controls and found that a rare, heterozygous missense variant in GDF15 (C211G) was highly enriched in HG cases versus controls^16^. However, to date, a mechanistic basis for these genetic associations has not been clearly elucidated. Here we demonstrate that GDF15 is truly elevated in NVP and HG and that the vast majority of GDF15 is of fetal origin. Remarkably, we show that rare and common genetic variants in *GDF15* that increase HG risk lower circulating GDF15 in the non-pregnant state, and that women with conditions that increase GDF15 before pregnancy state have lower incidence of NVP/HG; findings which appear to conflict with the known anorectic and emetic actions of GDF15. We resolve this apparent paradox by demonstrating that the anorectic actions of the GDF15-GFRAL axis are subject to desensitization and propose that antecedent levels of GDF15 influence maternal sensitivity to the surge of fetal derived GDF15 which occurs from early pregnancy onwards, thus determining the pregnant woman’s susceptibility to develop NVP and HG.

## Circulating GDF15 and severity of NVP

**Fig. 1:**
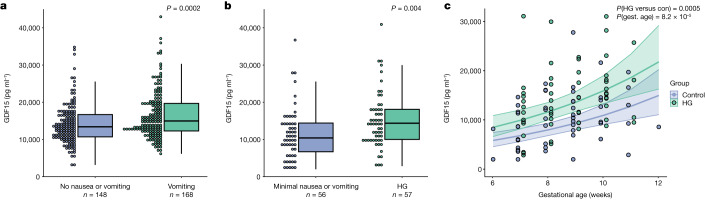
Circulating GDF15 is elevated in women experiencing nausea and vomiting in pregnancy and hyperemesis gravidarum. **a**, Dot and box plots illustrating the distribution of circulating GDF15 levels in women at approximately 15 weeks’ gestation with a history of vomiting in pregnancy versus those reporting no nausea and vomiting in pregnancy. *P* value is from an unadjusted linear regression model using natural-log transformed GDF15 concentrations. **b**, Dot and box plots illustrating the distribution of GDF15 levels (mean gestational age is approximately 10 weeks) in women presenting with hyperemesis gravidarum (HG) and those with low levels of nausea and vomiting in pregnancy. *P* value is from an unadjusted linear regression model using natural-log transformed GDF15 concentrations. **c**, Scatter plot illustrating the relationship between gestational age and GDF15 in the first trimester. The trend lines show predicted values of GDF15 levels (mean ± 95% CI) in women with and without HG from a linear regression model of natural-log transformed GDF15 with gestational age and HG status included as predictor variables. The *P* values are derived from the same regression model for the effect of HG (HG versus con) and gestational age (gest. age). Five participants (HG = 1, control = 4) included in the analysis in panel **b** are not plotted or included in this model as they were recruited after the first trimester. The box plots in **a** and **b** are Tukey box plots: the lower whiskers represent minimum values, the upper whiskers represent 1.5 × IQR and the upper and lower bounds of the box represent the 75th and 25th percentiles, respectively. The centre of the box represents the median. Source Data

A common genetic variant encoding amino acid residue 202 of GDF15 (H to D, hereafter H202D) that is associated with NVP and HG has recently been shown to systematically and substantially interfere with measurements of the peptide by reagents used in most of the studies that have reported GDF15 concentrations in human circulation^15^. We therefore commenced our investigations by measuring GDF15 in blood using an immunoassay that is less susceptible to confounding by the H202D variant (Supplementary Table 1); samples were taken at approximately 15 weeks’ gestation from women who completed a questionnaire relating to NVP. GDF15 levels were significantly higher in women reporting vomiting (*n* = 168) compared to those reporting no nausea or vomiting (*n* = 148) (Fig. 1a and Supplementary Tables 2–3). In a second study, we obtained blood samples from 57 women presenting to hospital with HG and from 56 controls who reported low levels of nausea and/or vomiting. Participants in each group were of similar age and body mass index and were predominantly in the first trimester of pregnancy when recruited (Supplementary Table 4). GDF15 levels (measured by an assay that is not susceptible to interference by H202D^15^) were significantly higher in women with HG versus those without (Fig. 1b,c and Supplementary Table 5). These results increase confidence that there is a true association between maternal GDF15 levels with HG and levels of nausea and vomiting in pregnancy.

## Origin of circulating GDF15 in pregnancy

**Fig. 2:**
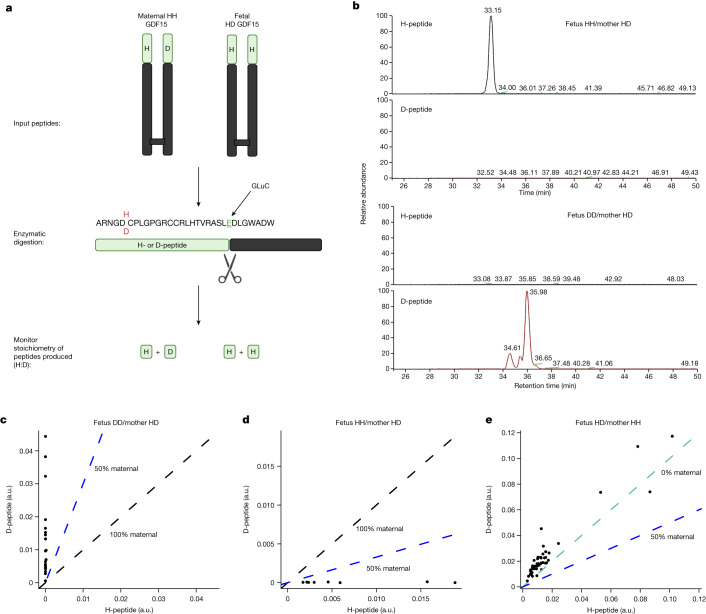
Circulating GDF15 in human pregnancy is predominantly of fetal origin. **a**, Schema of experimental design. The GDF15 dimer for maternal and fetal GDF15 is extracted and then digested with the endopeptidase GluC, cutting the N-terminal region into two distinct peptides with glutamic acid C-termini. The stoichiometry of the H- and D-peptides can then be monitored using liquid chromatography with tandem mass spectrometry to determine the relative levels of maternal or fetal derived GDF15 in the maternal circulation. **b**, Representative liquid chromatography–mass spectrometry retention time of H- and D-peptides from maternal plasma where the mother is heterozygous at H202D and the fetus is homozygous for the H or D allele as indicated. **c**–**e**, Scatter plots of the relative quantitation of H-peptide versus the D-peptide in plasma from pregnancies with the indicated genotypes. The dashed coloured lines indicate the expected relationships between the H- and D-peptides for the given circulatory origins of GDF15. **c**, *n* = 20 samples from five pregnancies. **d**, *n* = 8 samples from two pregnancies. **e**, *n* = 47 samples from 12 pregnancies. Panel **a** created using BioRender.com. a.u., arbitrary units. Source Data

**Extended Data Fig. 1:**
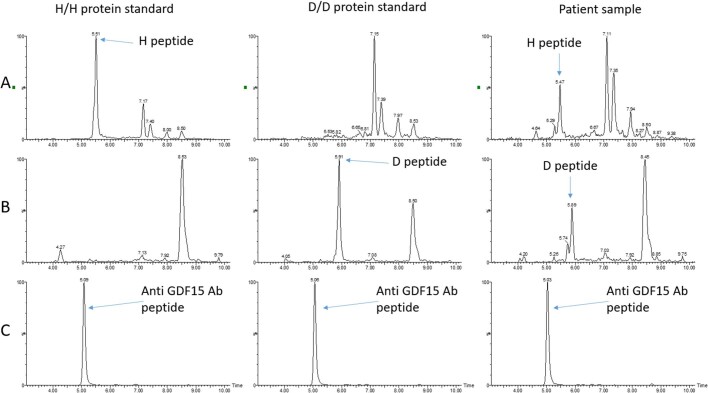
LC-MS/MS traces of two GDF15 related peptides and the murine anti-GDF15 antibody peptide from the heterozygous foetus analysis. **A**: N-terminal peptide from the wild-type protein, RT = ~ 5.47. **B**: N-terminal peptide from the mutant protein, RT = ~ 5.51. **C**: Peptide from the murine anti GDF15 antibody, RT = ~ 5.09. Data shown is traces generated from extracted plasma spiked with mutant homodimer, wild-type homodimer and an extracted participant sample.

**Extended Data Fig. 2:**
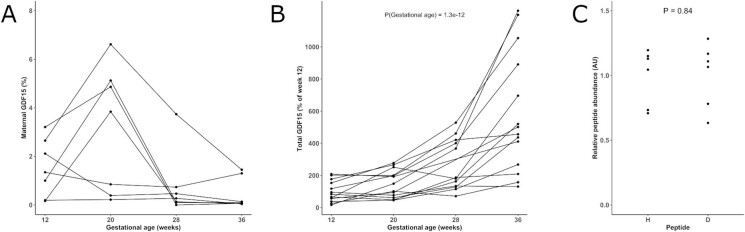
Measurement of foetal and maternally derived GDF15 in pregnancy. **A**: The estimated proportion of maternally derived GDF15 in 7 different pregnancies across 4 gestational ages where the foetus is homozygous for either the H or D at H202D and the mother is heterozygous at this site. **B**: The relative abundance of Total GDF15 measured by mass spectrometry in 14 different pregnancies where the foetus is homozygous for either H or D at H202D across 4 gestational ages, including the 7 genotype-discordant pregnancies presented in panel (A) and a further 7 pregnancies where the maternal genotype is concordant with the foetal genotype. Total GDF15 is expressed as a percentage of the mean value at 12 weeks gestation. Two sided P-value derived from a linear mixed model of log transformed Total GDF15~Gestational age with random intercepts. **C**: The relative abundance of N-terminal peptides from synthetic GDF15 homodimers with H or D at position 202 extracted using the R&D anti-GDF15 capture antibody coupled to magnetic beads. Plasma was fortified at the same concentration for each protein, extracted (n = 6) and analyzed by Orbitrap MS. AU = arbitrary units. Paired sample Wilcoxon test was used to compare the abundance of recovered H and D peptides, the presented P-value is two-sided. Source Data

GDF15 is widely expressed and, although the placenta is a site of high levels of expression, the relative contribution of the fetal and maternal tissues has not been established. To examine this, we developed mass spectrometry-based assays capable of distinguishing between GDF15 carrying a histidine or an aspartate at position 202 (position 6 in the mature circulating molecule) (Fig. 2a and Extended Data Fig. 1). Using placental RNA-seq data and maternal DNA from the Pregnancy Outcome Prediction (POP) study cohort^12^ we genotyped offspring and mothers (Supplementary Table 6) and studied seven H202D discordant offspring/mother pairs in which either the fetus or the mother alone was heterozygous at this site. Strikingly, in maternal plasma where the mother was heterozygous at H202D the discordant maternal peptide contributed, on average, to less than 1% of the total circulating GDF15 (median percentage D-peptide: 0.60% (Q1, Q3: 0.12, 2.25)) (Fig. 2b–d). The maternal fraction of GDF15 appeared to increase in some pregnancies between the first and second trimester but declined in later pregnancy (Extended Data Fig. 2a) as circulating concentrations of total GDF15 rise (Extended Data Fig. 2b). To confirm that antenatal circulating GDF15 was near-exclusively of fetal origin, we repeated these experiments using samples from maternal plasma where the fetus was heterozygous at the H202D position, and the mother was homozygous for the reference allele. Surprisingly the D-peptide, which is produced only by the fetus in this cohort, constituted more than half of the total circulating GDF15 (mean percentage D-peptide: 62.6%, 95% CI [59.1, 66.0], *P* = 6.80 × 10^−6^, one-sample *t*-test)—implying that it was present in excess of what would be expected even if all circulating GDF15 was fetal in origin (Fig. 2e). This was not attributable to assay bias (Extended Data Fig. 2c). These data suggest that the D-peptide may be preferentially expressed, secreted and/or may have a prolonged half-life in the circulation.

## Effects of a rare HG risk variant in GDF15

**Fig. 3:**
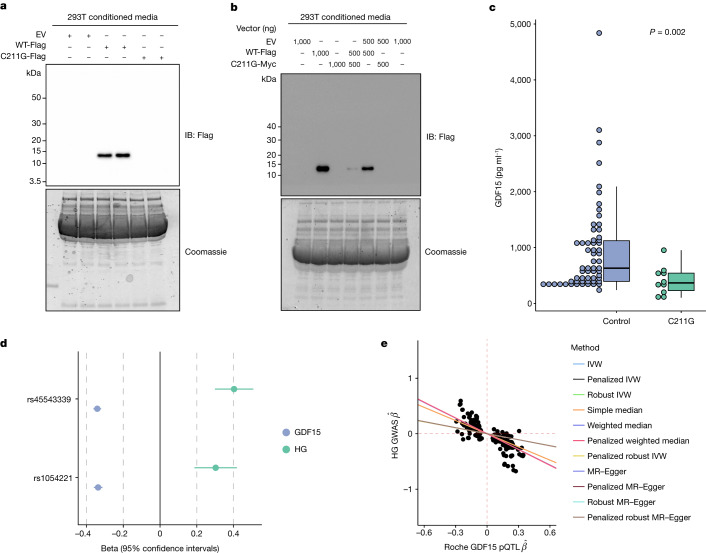
Rare and common hyperemesis gravidarum risk variants lower circulating GDF15 in the non-pregnant state. **a**, A rare HG risk variant, GDF15 C211G, impairs secretion of GDF15 as determined by western blotting of conditioned media from cells expressing Flag-tagged wild-type GDF15 (WT-Flag) or GDF15 C211G (C211G-Flag). **b**, GDF15 C211G impairs the secretion of wild-type GDF15 in a dominant-negative manner as co-expression of the mutant inhibited secretion of wild-type GDF15 from 293 T cells cotransfected with different amounts (shown in nanograms) of WT-Flag and Myc-tagged GDF15 C211G (C211G-Myc), as indicated. Representative images from three independent experiments are presented; EV = empty vector. **c**, Dot and box plots showing GDF15 levels measured using the Ansh total GDF15 assay in GDF15 C211G carriers (*n* = 10) identified by exome sequencing a Croatian population and age and sex-matched controls (*n* = 60) from the same study. *P* value is from a linear regression model of natural-log transformed GDF15 against C211G status. Box plot is a Tukey box plot: lower whiskers, minimum values; upper whisker (control), 1.5 × IQR; upper whisker (C211G), maximum value; box bounds, 25th, 50th and 75th percentiles. **d**, Forest plot illustrating the effect of HG risk SNPs (*n*(HG cases) = 1,306, *n*(controls) = 15,756) on circulating GDF15 measured in 18,184 participants in the Generation Scotland Study. Effect estimates for the rs1054221 variant are from an analysis conditioned on the lead HG variant rs45543339. The effect of the HG risk allele on circulating GDF15 in standard deviations and of the SNP on risk of HG in log-odds (±95% CIs) are shown. **e**, Scatter plot of HG genome-wide association study (GWAS) effect estimates (log-odds) versus Roche-based GDF15 pQTL effect estimates derived from *cis*-Mendelian randomization at the *GDF15* locus. Mendelian randomization was performed using 259 SNPs with genome-wide evidence of pQTL effects on GDF15 levels within 1 Mb *GDF15* locus and adjusted using LD estimates from UKBB (Methods). Causal effect estimates are reflected as regression lines. Source Data

**Extended Data Fig. 3:**
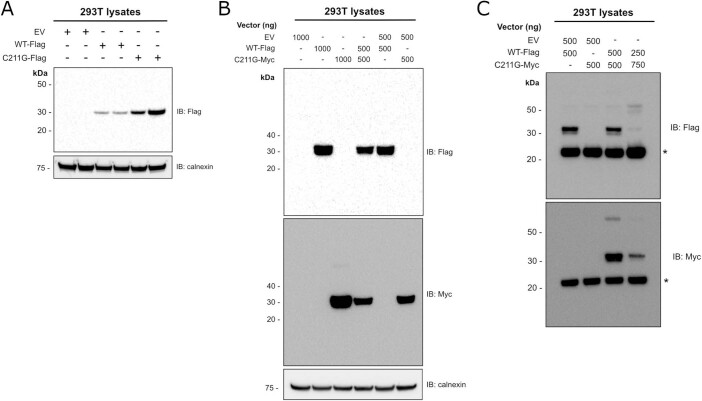
The C211G mutant is expressed intracellularly and heterodimerizes with its wild-type counterpart. **A**: Western blotting of cell lysates expressing Flag-tagged fusions of wild-type GDF15 (WT-Flag) or GDF15 C211G (C211G-Flag). **B**: Co-expression of wild-type GDF15 (WT-Flag) and Myc-tagged GDF15 C211G (C211G-Myc) does not impair the intracellular expression of wild-type GDF-15. **C**: WT and C211G form intracellular heterodimers, as judged by the co-immunoprecipitation of WT-Flag and C211G-Myc using anti-Flag antibodies. Asterisks mark co-eluted immunoglobulin light chains. Replicates, N = 3, representative images are shown. EV indicates transfections with the empty plasmid backbone only.

Fejzo et al. have previously reported that women heterozygous for the C211G mutation in GDF15 have at least a ten-fold increased risk of developing HG^16^. Cysteine 211 is one of the key conserved cysteine residues involved in intrachain disulfide bonding of GDF15 and its absence is predicted to be highly damaging^17^. Supporting this, when we transiently transfected a construct encoding GDF15 with a glycine at position 211 into HEK 293 T cells it was highly expressed but, unlike wild-type, the mature peptide was not secreted and the unprocessed pro-peptide was completely retained intracellularly (Fig. 3a and Extended Data Fig. 3a). GDF15 is secreted as a homodimer, so we wished to test whether the mutant form might interfere with the secretion of wild-type GDF15. We differentially tagged mutant and wild-type forms of GDF15 and demonstrated a clear reduction in the secretion of wild-type GDF15 when it was co-expressed with 211 G (Fig. 3b and Extended Data Fig. 3b,c).

To determine the effect of the C211G variant on circulating GDF15, we studied a Croatian cohort^18^ in whom exome sequencing had identified 11/2872 C211G heterozygotes (minor allele frequency of approximately 0.002). Levels of circulating GDF15 (measured by an in-house MSD assay using the Ansh Lab Total GDF15 antibodies) in C211G heterozygotes (none of whom were known to be pregnant) were reduced by more than 50% compared to age- and sex-matched controls from the same population (Fig. 3c and Supplementary Table 7).

To clarify the interaction between maternal and fetal carriage of the C211G variant, we identified 17 offspring of six women previously found to be heterozygous for C211G^16^. The mothers had HG in 10/10 pregnancies where the fetus was homozygous wild-type at position C211. Conversely, HG was reported in only 4/7 pregnancies where the fetus was heterozygous for C211G (Supplementary Table 8), suggesting that maternal carriage of the C211G variant confers HG risk and that this risk may be moderated when the variant is also carried by the fetus.

## Common HG risk variants and circulating GDF15

Common genetic variants in and around the *GDF15* gene have been reported to have the strongest genome-wide association with HG. We studied two single nucleotide variants at the *GDF15* locus which are independently associated with HG^3^ and examined their association with GDF15 levels (measured by Roche Elecsys) in 18,184 people from the Generation Scotland Study^19^. Consistent with the effects of the rare C211G variant, both HG risk alleles were associated with lower GDF15 in the non-pregnant state (rs45543339: *β* = −0.34 s.d., 95% confidence interval (CI) [−0.36, −0.32], rs1054221 conditioned on lead signal: *β* = −0.34 s.d., 95% CI [−0.36, −0.31]. Fig. 3d).

**Extended Data Fig. 4:**
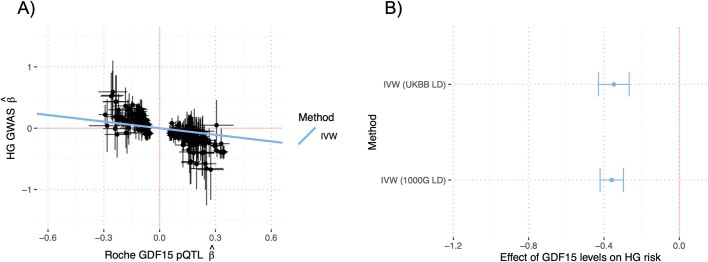
Mendelian Randomization estimates are robust to LD reference panel. MR was performed using m = 259 SNPs with genome-wide evidence of pQTL effects on GDF15 levels within 1 Mb GDF15 locus and adjusted using LD estimates from 1000 G WGS individuals (n = 489; see Methods). **A**: Scatterplot of HG GWAS effect estimates (ie log-odds) vs Roche-based GDF15 pQTL effect estimates. Vertical and horizontal lines represent 95% confidence intervals of HG effects and GDF15 effects, respectively. Causal effects were estimated using LD-aware IVW MR and depicted as a regression line. **B**: Forest plot of the IVW MR causal effect-size estimates of circulating GDF15 levels on HG risk from UK Biobank and 1000 G LD references. Each point represents the estimated causal effect and 95% confidence interval of a 1 standard deviation increase in circulating GDF15 in the non-pregnant state on HG risk in log-odds. The null of no mediating/causal effect is represented as a solid red line at 0.

**Extended Data Fig. 5:**
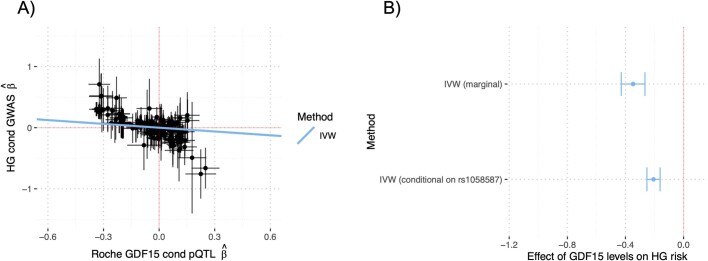
Mendelian Randomization estimates are robust to previously reported confounder SNP. MR was performed using m = 258 SNPs with genome-wide evidence of pQTL effects on GDF15 levels within 1 Mb GDF15 locus after residualizing (ie conditioning) on the effect of variant rs1058587, which was previously suggested to confound quantification of GDF15 levels^1,2^. Results were adjusted using LD estimates from UKBiobank WGS individuals (n = 138335; see Methods). **A** Scatterplot of conditional HG GWAS effect estimates (ie log-odds) vs conditional Roche-based GDF15 pQTL effect estimates. Vertical and horizontal lines represent 95% confidence intervals of HG effects and GDF15 effects, respectively. Causal effect estimates obtained using LD-aware IVW MR and reflected as regression lines. **B**: Forest plot of the causal effect-size estimates of circulating GDF15 levels on HG risk from standard (ie marginal) pQTL/GWAS results and those obtained using pQTL/GWAS results conditioned on variant rs1058587. Each point represents the estimated causal effect and 95% confidence interval of a 1 standard deviation increase in circulating GDF15 in the non-pregnant state on HG risk in log-odds. The null of no mediating/causal effect is represented as a solid red line at 0.

To systematically test for a causal relationship between circulating GDF15 in the non-pregnant state and HG risk, we performed linkage disequilibrium (LD)-aware Mendelian randomization analysis using *cis*-protein quantitative trait loci (pQTLs) (*P* < 5 × 10^−8^) identified in a genome-wide association study of circulating GDF15 (measured by Roche Elecsys) in the Generation Scotland Study (*n* = 18,184). Overall, we observed that increased circulating GDF15 in the non-pregnant state reduced HG risk (inverse variance weighted MR; OR = 0.70 per s.d. increase in GDF15; 95% CI [0.65–0.76], *P* = 6.98 × 10^−17^) (Fig. 3e and Supplementary Table 9). These results were robust to the choice of LD reference panel, instrument selection, and Mendelian randomization approach (Extended Data Fig. 4 and Supplementary Table 9–11). While we have previously demonstrated that the Roche Elecsys assay is not affected by the common protein altering variant H202D (rs1058587)^15^, we wished to exclude any possibility that small biases in detection related to this variant could explain our findings. Therefore, we repeated our analysis after conditioning on this variant and found similar results (Extended Data Fig. 5 and Supplementary Table 12).

Finally, we used statistical colocalization implemented in coloc^20^, a complementary approach to Mendelian randomization, which can be used to assess the probability that a genetic signal is shared between an outcome of interest and an intermediate molecular trait, in this case HG and circulating GDF15, respectively. We observed two colocalizing signals at the *GDF15* locus (rs45543339 and rs1227731; PPH4 > 0.99, Supplementary Table 13), which correspond to the two independent signals presented in Fig. 3d, where both HG risk-raising alleles were associated with reduced GDF15 in the non-pregnant state.

Thus, from studies of both rare and common genetic variants in GDF15, it appears that higher circulating levels of the hormone in the non-pregnant state are associated with protection from HG.

## Evidence for desensitization to GDF15

**Fig. 4:**
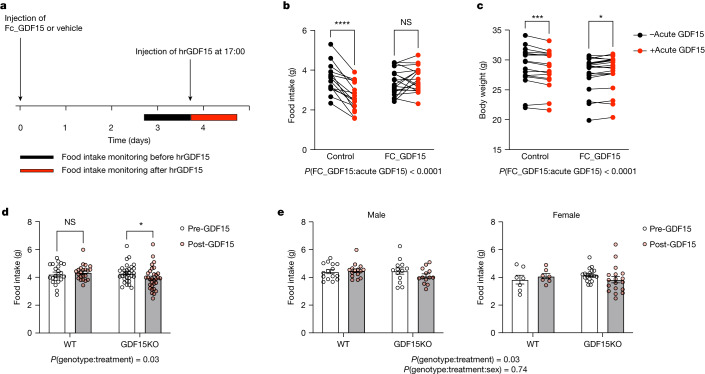
Treatment with long-acting GDF15 influences the response to the anorectic actions of acute GDF15 treatment in mice. **a**, Schema of the experimental paradigm for results presented in **b** and **c**. Adult male and female C57Bl/6 J mice were injected with 0.01 mg kg^−1^ of Fc-GDF15 fusion protein (Fc_GDF15) or vehicle control (PBS). Food intake was measured overnight (from 17:00 to 09:00) before (black bar) and after treatment (red bar) with short acting human recombinant GDF15 (hrGDF15 0.1 mg kg^−1^). **b**, Food intake recorded overnight (17:00 to 09:00) the day before (black dots) and after an acute bolus of hrGDF15 (red dots) in mice with and without pretreatment with Fc_GDF15. **c**, Body weight at 09:00 the day before (black dots) and 09:00 the day after (red dots) an acute bolus of hrGDF15 in mice with and without pretreatment with Fc_GDF15. *n* = 17 (12 male, 5 female) in the control and 19 in the FC_GDF15 group (13 male, 6 female). **d**–**e**, In the wild-type (*n* = 22, 7 female, 15 male) and *Gdf15*^*−/−*^ (*n* = 31, 13 male, 18 females, GDF15KO) mice food intake was measured for 24 h before (Pre-GDF15) and after (Post-GDF15) treatment with 0.01 mg kg^−1^ human recombinant GDF15. **d**, Mean ± s.e.m. food intake over a 24 h period before and after GDF15 treatment. **e**, The same data (mean ± s.e.m. food intake) is plotted with individual data points disaggregated by sex to demonstrate the consistency of the effect across both sexes. All *P* values presented in this figure are two-sided. Data were analysed with mixed-effects models, post hoc testing comparing food intake and body weight before and after acute GDF15 treatment was conducted with the Sidak test to correct for multiple testing. **b**–**c**, *P = 0.02, ***P = 0.0006, ****P < 0.0001. **d**, *P = 0.03. NS, not significant. Source Data

**Extended Data Fig. 6:**
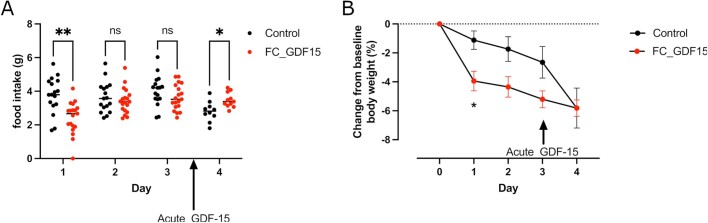
Longitudinal effects of long acting GDF15 on food intake and body weight and mice. **A**-**B**: The effects of 0.01 mg/kg of Fc-GDF15-15 fusion protein (FC_GDF15) or vehicle control (PBS) on food intake (**A**) or body weight (**B**). In (A) Days 1 – 3 represent 24-hour food intake from 5 pm to 5 pm after treatment with control or FC_GDF15. Day 4 represents food intake from 5 pm to 5 pm after both groups received an acute bolus of human recombinant GDF15 (0.1 mg/kg). The black lines indicate mean food intake for each day. In (B) mean +/− SEM change in body weight at 5 pm is presented as a percentage of baseline body weight. Days 1–3: N = 17 (12 male, 5 female) in Control and 19 in FC_GDF15 group (13 male, 6 female). Day 4: N = 10 (5 male, 5 female) in Control and 11 in FC_GDF15 group (5 male, 6 female) – as one cohort of mice were sacrificed at 9am on Day 4. Hypothesis testing was conducted using a mixed-effects model. Post-hoc testing comparing Control and FC_GDF15 treated groups was undertaken with the Sidak test to correct for multiple testing. **A**: **P = 0.002,*P = 0.04, ns = non-significant, P > 0.05, **B**:*P = 0.02. All reported P-values are two-sided. Source Data

To test the hypothesis that prior levels of exposure could influence acute responses to GDF15 we administered a long-acting form of GDF15 (human FC_GDF15, 0.01 mg kg^−1^)^21^ to wild-type mice (Fig. 4a). Pretreatment with FC_GDF15 resulted in a mean concentration of 4,773 ± 440 pg ml^−1^ three days after the injection, which corresponds to an approximate 47-fold increase compared to basal circulating levels of mouse GDF15. This resulted in transient suppression of food intake for one day after injection relative to untreated controls (Extended Data Fig. 6). Three days after treatment with FC_GDF15, mice were then given an acute bolus of human recombinant GDF15 (0.1 mg kg^−1^), which typically elevates GDF15 to greater than 20,000 pg ml^−1^ 1 h after injection^22^, and its effects on food intake and body weight were measured. Mice previously receiving a vehicle control injection showed the expected major reduction in food intake in response to a bolus of GDF15 (Fig. 4b) and lost weight (Fig. 4c). In contrast, mice previously exposed to GDF15 had a markedly blunted acute response (Fig. 4b,c), supporting the notion that elevated antecedent levels of GDF15 can influence the subsequent action of an acute rise in circulating GDF15. To independently examine whether basal GDF15 levels can modulate the anorectic actions of acute GDF15 administration, we studied mice congenitally lacking GDF15 (*Gdf15*^*−/−*^) treated with a dose of human recombinant GDF15 (0.01 mg kg^−1^) that, when given to wild-type mice, does not typically reduce food intake over a 24 h period. We compared the effects of this dose of GDF15 in G*df15*^*−/−*^ mice and their wild-type littermates. Then, 24 h after GDF15 administration, food intake was suppressed in GDF15-deficient mice but not in their wild-type littermates (Fig. 4d,e). Together, these orthogonal experiments establish that the anorectic actions of acute GDF15 can be modulated by prior GDF15 exposure.

## Chronic GDF15 elevation and NVP risk

Some chronic medical conditions are characterized by life-long elevations of GDF15. Our hypothesis predicted that such exposure might reduce the risk of developing nausea and vomiting when those individuals become pregnant. Beta-thalassaemia is a genetic disorder affecting red blood cells where extremely high levels of GDF15, found throughout life^4,5^, are thought to come from the expanded mass of stressed erythroblasts. Though fertility is impaired in this disease, some women, particularly those with thalassaemia intermedia, do become pregnant. We conducted a survey (Supplementary Information) of women with β-thalassaemia who had undergone at least one pregnancy that had resulted in a live birth and compared the results with those from ethnically and age-matched women who did not have thalassaemia. There was a strikingly lower prevalence of symptoms of NVP in the women with thalassaemia: only approximately 5% of women with thalassaemia reported any nausea or vomiting compared to greater than 60% of the controls (*P* < 0.01) (Supplementary Table 14).

## Summary and conclusions

Despite the fact that nausea and vomiting are symptoms which occur in most human pregnancies, are commonly disabling and, when severe, can be life-threatening^2^, their aetiology and pathogenesis have remained poorly understood. Here we present evidence that the severity of nausea and vomiting of pregnancy is the result of the interaction of fetal derived GDF15 and the mother’s sensitivity to this peptide, which is substantially determined by her prior exposure to the hormone.

Using immunoassays that are not confounded by the common H202D variant we showed that levels of GDF15 in the maternal circulation in the late first trimester are significantly higher in women with HG compared to those without severe nausea and vomiting. Similarly, in an independent cohort of pregnant women, circulating GDF15 measured in the second trimester was found to be elevated in participants who reported vomiting in pregnancy compared to those who reported no nausea or vomiting. We can now conclude with confidence that higher circulating levels of GDF15 in maternal blood are associated with an increased risk of NVP and HG. However, as there is considerable overlap in levels between HG cases and controls, GDF15 concentrations alone cannot be used as a diagnostic tool to differentiate HG from other causes of vomiting in a pregnant woman.

We applied mass spectrometry to genetically discordant mother/offspring pairs and identified the feto-placental unit as the predominant source of GDF15 circulating in maternal blood. This finding is consistent with previous reports of extremely high levels of *GDF15* gene expression in, and protein secretion from, human trophoblasts^11^. A caveat to this observation is that these studies were undertaken in healthy pregnancies, and it is conceivable that, in women with established HG, stressed maternal tissues may, in theory, make an additional contribution to the circulating pool.

The rare coding variant GDF15 C211G has been reported to greatly increase the risk of HG^16^. We report that this mutation is associated with markedly lower circulating levels in the non-pregnant state attributable to the deleterious effects of the mutation on secretion of mature GDF15, including any wild-type subunit present in heterodimeric GDF15. We also demonstrate that common HG risk conferring variants are associated with lower circulating levels of GDF15 in the non-pregnant state. Conversely, high levels of GDF15 preceding pregnancy, as are found in thalassaemia, are associated with a reduction in the prevalence of NVP. This finding is consistent with studies which report that prepregnancy cigarette smoking, a behaviour associated with elevated GDF15 (ref. ^23^), reduces the risk of HG^24^.

Agonist-induced desensitization is a feature of many hormone-receptor systems and here we show that this may occur in the case of GDF15, and its receptor GFRAL-RET. Mice exposed to mildly supraphysiological doses of GDF15 for three days show markedly attenuated food intake and body weight responses to an acute bolus of GDF15. This apparent tendency for the GDF15/GFRAL-RET system to exhibit some degree of desensitization provides a plausible explanation for the effects of prepregnancy GDF15 exposure on the risk of NVP and HG developing in the face of the acute increase in circulating GDF15 which begins in early pregnancy.

We report that levels of GDF15 are higher in pregnant women with NVP and HG than in those without these symptoms and have also shown that the feto-placental unit is the major source of that GDF15 in maternal blood. Mothers with HG are enriched in GDF15 variants which are associated with lower GDF15 in the non-pregnant state and will transmit approximately 50% of those alleles to their offspring, in whom they might be expected to lower GDF15 levels. How can those observations be reconciled? First, it is possible that variants that affect the expression of GDF15 do so differentially in adult tissue versus placenta. Second, there are factors beyond the *GDF15* gene which may influence GDF15 production by the feto-placental unit. For example, female fetal sex, the presence of twins or the presence of invasive trophoblastic disease are all associated with increased HG risk^25,26^ and, at least in the case of female fetuses, increased GDF15 levels in pregnancy^27^. In the case of the C211G HG risk variant, we found suggestive evidence for an interaction between maternal and fetal GDF15 genotypes, with fetal carriage of this variant apparently moderating the maternal effect. Thus, HG occurred in ten out of ten pregnancies where the mother was a C211G heterozygote (presumably with low prepregnancy levels of circulating GDF15) and was carrying a wild-type fetus, but only in four out of seven pregnancies where the fetus was heterozygous for the mutation. Given the small sample size these results should be considered hypothesis generating and require replication in larger studies with maternal and fetal genotype, HG symptoms and antenatal GDF15 measurements.

Our findings have obvious implications for the prevention and treatment of HG. The acute rise in GDF15 which accompanies normal pregnancy is, we would argue, likely to be necessary, if not sufficient, for the causation of HG. The corollary of this is that blocking GDF15 action in the pregnant mother should be a highly effective therapy for women suffering from HG. We make this argument based on a number of observations. First, the administration of an acute bolus of GDF15 to humans, resulting in levels similar to that seen in pregnancy, frequently results in nausea and vomiting^8^. Second, in non-human primates, blocking GDF15 is highly effective in reducing vomiting resulting from the administration of drugs such as *cis*-platinum which cause an acute increase in GDF15 (ref. ^9^). Third, human genetic variants, common and rare, point to the GDF15 system as a major susceptibility locus for human HG^3,16^. Fourth, the striking reduction in frequency of NVP in women with thalassaemia, a condition of markedly increased prepregnancy levels of GDF15, suggests that GDF15 plays a key role in the causation of these symptoms in pregnancy. The fact that high GDF15 levels in the non-pregnant state appears to protect against the development of NVP and HG suggests that strategies which safely increase circulating GDF15 levels before pregnancy may be useful in the prevention of these conditions. The safety of recombinant GDF15, at least in the short term and outside of pregnancy, has been demonstrated in Phase 1 clinical trials and this could be administered at low doses before pregnancy with the intention of inducing GDF15/GFRAL desensitization^4^. Alternatively, metformin robustly increases GDF15 in humans^28^ and could be tested as a prophylactic agent for HG. While metformin is often prescribed off-label in the periconception period in polycystic ovarian syndrome where it may improve fertility, possible adverse effects on fetal growth have been described when it is used in the context of gestational diabetes^29^ and these should be borne in mind when evaluating its potential safety and efficacy. Regardless of specific agents used, more information on the dose-response and time course of GDF15 desensitization in humans is required before planning trials of prepregnancy GDF15 exposure in women at risk of HG.

Since the tragedy of thalidomide^30^, concerns about safety have understandably been very prominent in discussions of new treatments for HG, particularly any that would cross the placenta and carry a risk of teratogenesis. For other disease indications, antibodies have been engineered to minimize their transplacental passage, and have been widely used^31^, so this should be a possible route to safe blockade of GDF15 signalling. There are reasons to think that highly specific blockade of GDF15 signalling through its receptor GFRAL is likely to be safe, even if such an antagonist did gain access to the fetus. GDF15 appears to act specifically through GFRAL, which is expressed only in the hindbrain. Mice lacking GDF15 or GFRAL develop normally and remain largely healthy throughout life.

GDF15 appears to have evolved primarily as a signal to confer information about a range of somatic stresses to the brain to reduce continuing exposure to those stresses at the time of exposure and in the future, through promoting avoidance behaviour^1^. The placentae of certain higher mammals, including primates, have evolved to produce large amounts of GDF15 from early pregnancy, a phenomenon which likely explains the very common occurrence of nausea and vomiting in pregnant women^11^. Sherman and Flaxman^32^ suggested that the evolutionary basis for this likely lay in the protection of both mother and fetus from food-borne illness and toxins, particularly important at a time when the fetus is most susceptible to teratogens and the immunosuppressed state of early pregnancy makes mothers susceptible to infections. The energy needs of the growing fetus may outweigh those risks as the pregnancy progresses resulting in the selection against persistence of NVP beyond the 1st trimester in normal pregnancies. The phenomenon of ligand induced desensitization, which we have demonstrated to occur with GDF15 may explain the natural tendency for the severity of NVP to wane as pregnancy progresses.

Our work has some important limitations. Our Mendelian randomization estimates did not account for fetal genotype which is 50% correlated with maternal genotype and GDF15-raising alleles may also be functional in placenta. However, given the divergent effects of maternal and fetal GDF15 on HG risk—our estimates may actually be an underestimate of the protective effects of prepregnancy GDF15 elevation. While we have provided two orthogonal pieces of evidence that prior exposure to GDF15 alters the food intake response to a subsequent bolus of GDF15, further work is required to better understand the level at which such modulation occurs and to experimentally establish that desensitization occurs to the nauseating and aversive effects of GDF15 in longer-term animal experiments and in humans. Finally, while our work establishes a significant role for GDF15 in the pathogenesis of NVP and HG, it may have other roles in maternal and fetal health which have not yet been fully explored.

In conclusion, our findings place GDF15 at the mechanistic heart of NVP and HG and clearly point the way to strategies for its treatment and prevention.

## Methods

### Cambridge Baby Growth Study

The Cambridge Baby Growth Study (CBGS) is a prospective, longitudinal cohort study originally recruiting 2,229 pregnant women from the Rosie Maternity Hospital, Cambridge between April 2001 and March 2009 (ref. ^13^). This analysis was performed using a nested case–control format from those women who returned filled-in pregnancy questionnaires, including questions about nausea and vomiting in pregnancy, and who provided a blood sample between 12 and 18 weeks of pregnancy. The cases were women who reported vomiting in pregnancy, and the controls were women who reported neither nausea nor vomiting in pregnancy. The samples for GDF15 measurement were chosen according to availability. The statistical analysis was performed using linear regression (and natural-log transformed GDF15 concentrations so that the residuals were normally distributed), either unadjusted or adjusted for potential confounders such as gestational age at sampling and the body mass index. Ethical approval for the Cambridge Baby Growth Study was granted by the Cambridge Local Research Ethics Committee, Cambridge University Hospitals NHS Foundation Trust, Cambridge, UK (00/325). Written informed consent was obtained from all the study participants.

### HG study

The HG Study is a case–control study of women recruited from the Rosie Maternity Hospital, Cambridge and North West Anglia (NWA) NHS Foundation Trust at Peterborough City Hospital, between 2018 and 2021. The 72 cases were pregnant women admitted to hospital for rehydration due to hyperemesis gravidarum. The 182 controls were pregnant women admitted to hospital in the same pregnancy timeframe as the cases, but for other reasons (for example, termination of pregnancy or uterine bleeding). Blood samples were collected around week nine of pregnancy, and a nausea/vomiting score was calculated by asking the women for their current and worst nausea and vomiting ratings out of ten. The samples for GDF15 measurement were chosen to maximize the difference in the nausea/vomiting scores. The statistical analysis was performed using linear regression (and natural-log transformed GDF15 concentrations so that the residuals were normally distributed), either unadjusted or adjusted for potential confounders such as gestational age at sampling. Ethical approval was granted by the National Research Ethics Service Committee for East of England, Norfolk, UK (14/EE/1247). All procedures followed were in accordance with both institutional and international guidelines. Written informed consent was obtained from all women.

### C211G carriers and controls in the CROATIA-Korcula study

The CROATIA-Korcula study sampled 2,926 Croatians from the Adriatic island of Korcula between the ages of 18 and 98. The fieldwork was performed from 2007 to 2014. Ethical approval was given for recruitment of all participants by ethics committees in both Scotland and Croatia. All volunteers gave informed consent before participation. Carriers of GDF15 C211G variant with available serum samples were identified from the exome-sequence of samples from the CROATIA-Korcula study and were paired with age- and sex-matched controls from the same cohort.

### Common genetic variation, circulating GDF15 and risk of hyperemesis gravidarum

#### 23andMe HG GWAS data

We obtained 23andMe, Inc. (23andMe) GWAS summary statistics of HG from ref. ^3^. Briefly, 23andMe GWAS research participants provided answers to morning sickness-related questions. All research participants provided informed consent and volunteered to participate in the research online, under a protocol approved by the external AAHRPP-accredited Institutional Review Board (IRB), Ethical & Independent Review Services. As of 2022, the Ethical & Independent Review Services is part of Salus IRB (https://www.versiticlinicaltrials.org/salusirb). HG status was defined as 1,306 research participants who reported via an online survey that they received intravenous therapy for NVP and 15,756 participants who reported no NVP served as controls. For additional details refer to ref. ^9^.

#### GDF15 pQTL data and quality control

Generation Scotland is a family- and population-based study consisting of 23,690 participants recruited via general medical practices across Scotland between 2006 and 2011. The recruitment protocol and sample characteristics are described in detail elsewhere^33,34^. Ethical approval for the Generation Scotland study was obtained from the Tayside Committee on Medical Research Ethics (on behalf of the National Health Service).

The GWAS analysis used BOLT-LMM to adjust for population structure and relatedness between individuals^35^ in a linear mixed model analysis of Generation Scotland participants with available GDF15 data and Haplotype Reference Consortium reference panel release 1.1 (refs. ^36,37^) imputed genotype information (18,184 individuals). Age, sex and first 20 principal components were included as covariates. Serum GDF15 concentrations were subject to rank-based inverse normal transformation before analysis. Associations were considered significant when *P* ≤ 5 × 10^−8^. Full details of quality control and preparation of the imputed genotype data are available^37^.

#### Conditional GWAS analyses

To assess the extent to which signals beyond lead (or focal) SNPs contribute to either HG risk (or GDF15 levels), we performed conditional analyses using GWAS summary data and estimates of LD derived from the regression of the summary statistics model (namely, RSS)^38^. Briefly, given estimated effect sizes *β* (for example, log-odds or linear effects) at *m* non-leading SNPs, corresponding *m* standard errors *s*, *m* *×* *m* LD matrix *V* and *m* *×* 1 vector **v** (transpose **v**^T^) of LD estimates with the lead SNP, we can compute residual effect sizes ${\beta }^{* }$ as

$${\beta }^{\ast }|z\approx N(\beta -S{\bf{v}}z,S(V-{\bf{v}}{{\bf{v}}}^{{\rm{T}}})S)$$

where *S* *=* diag(*s*) is the *m* *×* *m* diagonal matrix of standard errors, *z* *=* *b*/s.e.(*b*) is the association statistic at the lead (or focal) SNP and *N*(⋅,⋅) corresponds to the multivariate normal distribution. The conditional estimates correspond to the mean of the above distribution and standard error proportional to the diagonal of the covariance.

To compute conditional effect-size estimates for circulating GDF15 levels and separately for HG risk, we used the above model focusing on *m* = 310 harmonized variants and LD estimates from WGS data in European-ancestry individuals in the UK Biobank cohort (see below).

#### Mendelian randomization analyses

To perform Mendelian randomization between circulating GDF15 levels with HG risk, we harmonized Roche-based GDF15 pQTL, GWAS and LD reference panels to obtain valid estimates. First, we restricted analysis to variants associated with GDF15 levels at a genome-wide significant threshold (*P* < 5 × 10^−8^) ± 500 kb around the transcription start site. Next, we harmonized GDF15 pQTL significant results with 23andMe HG GWAS association statistics to match for consistent reference and alternative alleles, which resulted in *m* = 311 variants. We excluded any variants whose reference and alternative alleles may be ambiguous (for example, G/C, A/T), except for previously referenced risk alleles (for example, rs1058587). To account for linkage between GDF15-associated variants, we estimated LD using WGS data from European-ancestry individuals in the UK Biobank (UKBB) cohort (*n* = 138,355) as well as WGS data from European-ancestry individuals in the 1000 G study (*n* = 489). To derive LD estimates in UKBB the publicly available whole-genome sequencing (WGS) data from European participants in UKBB (*n* = 138,335) was used for the determination of linkage disequilibrium at the GDF15 locus. A total of 5,259 WGS variants were extracted ± 500 kb from chr. 19: 18388612:C:G (GRCh38) and Pearson’s *R* was determined using the PLINK v.1.90b6.26/Swiss Army knife App via the UKB Research Access Platform, with the following parameters ‘--ld-window-r2 0 --ld-window 10000 --keep-allele-order --snp chr19:18388612:C:G --window 1000’. All work using the UKBB resource was conducted using application numbers: 9905 and 32974.

Harmonizing our association data with LD estimates resulted in *m* = 259 variants for UK Biobank data and *m* = 310 variants when using 1000 G data. Lastly, we performed Kriging analysis using the R package susieR (https://cran.r-project.org/web/packages/susieR/) to ensure no variants were mislabelled between reference LD and association study results. Finally, to perform Mendelian Randomization, we used the R package MendelianRandomization (https://cran.r-project.org/web/packages/MendelianRandomization/index.html). Briefly, the Mendelian randomization approach models a relationship between inferred effect sizes between exposure (circulating GDF15 levels) and outcome (HG risk). Specifically,

$${\widehat{\beta }}_{{\rm{hg}}}\approx N(V\,{\widehat{\beta }}_{{\rm{GDF}}15}\alpha ,SVS)$$

where ${\widehat{\beta }}_{{\rm{hg}}}$ refers to estimated log-odds from HG GWAS, ${\widehat{\beta }}_{{\rm{GDF}}15}$ are estimated effect sizes of circulating GDF15 levels, *α* is the putative causal effect, *S* is the diagonal matrix of HG standard errors and *V* is the LD matrix. We perform inference of *α* using instruments (that is, GDF15 variants) selected through genome-wide significance (*P* < 5 × 10^−8^), as well as variants found in susieR credible sets (namely, set of variants with cumulative posterior probability to explain GDF15 associations greater than 0.95, rs11881403, rs888663 and rs16982345, rs1227734).

#### Colocalization analyses

To perform colocalization analysis between genetic variants underlying circulating GDF15 levels and HG risk, we performed the same harmonization strategy as the LD-aware Mendelian randomization analysis in Roche-based GDF15 pQTL data and 23andMe GWAS results. However, rather than limit analyses to variants with genome-wide significance for pQTL effects, we selected all variants represented in LD estimated from UK Biobank WGS data, which resulted in *m* = 2,297 variants. We performed multi-causal SNP colocalization using the R package coloc (https://cran.r-project.org/web/packages/coloc/index.html), which tests for colocalization across SNPs identified within credible sets, to better reflect linkage patterns.

### Prevalence of nausea and vomiting in pregnancy in thalassaemia

We conducted a survey to compare the prevalence of NVP among women with β-thalassaemia and ethnically and age-matched healthy women at the Colombo North Teaching Hospital, Ragama, Sri Lanka from 1 June to 31 August 2022. All female patients with β-thalassaemia with at least a single child attending for regular blood transfusions and thalassaemia follow-up during the study period were recruited. An equal number of ethnically and age-matched healthy women without thalassaemia with at least a single child attending the general paediatric clinic of the same hospital with their children during the study period were recruited as controls. Specifically, we recruited the eligible ethnically and age-matched control participant without thalassaemia attending the clinic on the same day immediately after recruiting a participant with β-thalassaemia. Informed written consent was obtained from all study participants before recruitment. Data on nausea, vomiting and loss of appetite during pregnancy were gathered using an interviewer-administered questionnaire (Supplementary Information). The prevalence of nausea, vomiting and loss of appetite during pregnancy of participants with and without β-thalassaemia were compared using logistic regression after adjusting for parity, number of children and time since index pregnancy. The study was approved by the Ethics Review Committee of University of Kelaniya, Sri Lanka (reference no. P/228/11/2019).

### Maternal NVP levels and offspring genotype

Carriers of rs372120002 (C211G) were identified in a previous whole-exome sequencing study of hyperemesis gravidarum^16^. Eleven carriers of rs372120002 (C211G) and their children were invited to participate in the offspring study, among which six carrier mothers and 17 children agreed to participate. Participating mothers filled out a survey on NVP/HG during each of their pregnancies which included whether they had HG, were treated with antiemetic medication(s) and intravenous fluids, had an emergency room visit and/or hospitalization for HG and when their symptoms resolved. Cheek swab samples were collected from children using DNA Genotek cheek swab kits (OCD-100, OC-175, Oragene), and DNA was extracted according to the manufacturer’s recommendations. Polymerase chain reaction (PCR) of rs372120002 was performed using standard methods with forward primer CAGCTCAGCCTTGCAAGAC and reverse primer GGATTGTAGCTGGCGGGC, annealing temperature at 60 °C, and the PCR product was sequenced by Azenta, Life Sciences. Genotypes were called using the 4Peaks app to view DNA trace files. The study was approved by the University of Southern California IRB.

### GDF15 immunoassays

Total GDF15 levels in the CBGS cohort were measured using a three-step plate ELISA (Ansh AL-1014-r) which was validated to be able to recognize H- and D-containing variants at position 202 (position six of the mature peptide) of GDF15 with comparable affinity (Supplementary Table 1). The calibrators, kit controls, in-house sample pool controls (diluted 1:15 in Sample Diluent) and samples (diluted 1:15 in Sample Diluent) were added to the antibody coated microtiter plate and incubated. Following a wash step the biotinylated detector antibody was added and incubated. Following a second wash step streptavidin horse radish peroxidase conjugate solution was added and incubated. Following a third wash step substrate solution (TMB) was added and incubated followed by an acidic stop solution. The measured absorbance at 450 nm corrected at 630 nm is directly proportional to the GDF15 concentration. The calibrator supplied with the kit by Ansh Labs is traceable to recombinant human GDF15 from R&D Systems (Bio-Techne). Ansh Lab ELISA Total GDF15 between batch imprecision kit controls 7.7% at 173.2 pg ml^−1^, 5.1% at 480.0 pg ml^−1^ and in-house sample pool controls 4.9% at 397.5 pg ml^−1^, 3.7% at 1,022.5 pg ml^−1^.

GDF15 measurements in both the HG versus control study and in Generation Scotland were measured on a Cobas e411 analyser (Roche Diagnostics) using the manufacturer’s reagents and quality control material. The coefficient of variation for GDF15 was 3.8% for the low control (at 1,556 pg ml^−1^) and 3.4% for the high control (at 7,804 pg ml^−1^). The limit of detection of the GDF15 assay is set to 400 pg ml^−1^ by the manufacturer, and the upper limit of the measuring range was 20,000 pg ml^−1^. As previously reported^19^ for the Generation Scotland study, for continuous analysis, samples below the limit of detection were reported as 200 pg ml^−1^ and samples above the measuring range as 25,000 pg ml^−1^. For the HG versus control pregnancy study, samples were diluted 1 in 5 with assay buffer before measurement because of the known very high levels in pregnancy. To examine the effect of the H202D variant on GDF15 immunoreactivity we determined the recovery of synthetic peptides produced as previously described^15^.

Total GDF15 levels in the Croatia-Korcula study were measured using an in-house assay developed on the Meso Scale Discovery (MSD) platform using two monoclonal antibodies from Ansh Labs which have been described as being able to recognize H- and D-containing variants at position 202 (position six of the mature peptide) of GDF15 with comparable affinity. The calibrators, in-house sample pool controls and samples were added to the monoclonal antibody-coated MSD plate and incubated. Following a wash step the biotinylated detector monoclonal antibody diluted in MSD Diluent 100 was added and incubated. Following a second wash step Sulpho-TAG labelled Streptavidin (MSD) diluted in MSD Diluent 100 was added and incubated. Following a third wash step MSD read-buffer was added to all the wells and the plate was immediately read on the MSD s600 plate reader. Luminescence intensities for the standards were used to generate a standard curve using MSD’s Workbench software package and were directly proportional to the GDF15 concentration. The calibrator is recombinant human GDF15 from R&D Systems (Bio-Techne). MSD Ansh antibody Total GDF15 between batch imprecision based on in-house sample pool controls 10.2% at 552.4 pg ml^−1^, 11.7% at 1,518.6 pg ml^−1^ and 11.7% at 7,036.1 pg ml^−1^.

### Identification of mother/fetus pairs discordant for H202D

Mother–offspring pairs not fully concordant for genotype at the H202D site in GDF15 were identified by first genotyping the offspring using placental RNA sequencing data from the Pregnancy Outcome Prediction study cohort^12^. We used the GATK pipeline^39^ to identify SNPs from the RNA-seq alignment data (that is, BAM files). Briefly, the pipeline comprises the following steps: (1) marking duplicate reads using ‘markDuplicate’ of Picard (https://broadinstitute.github.io/picard/), (2) splitting reads that contain ‘N’s in their CIGAR string using ‘splitNRead’ of GATK (subsequent submodules from GATK hereafter), (3) realignment of reads around the indel using ‘IndelRealigner’, (4) recalibrating base quality using ‘BaseRecalibrator’ and (5) calling the variants using ‘HaplotypeCaller’. Homozygous alternative alleles and their read counts were parsed directly from the VCF files generated by step (5). As homozygous reference alleles are not called by ‘HaplotypeCaller’, we used ‘mpileup’ command of samtools and bcftools to detect the read counts from the BAM files generated by the previous step. For heterozygous SNPs, we counted reads by the reference and alternative bases using ‘ASEReadCounter’. Fetal genotype was confirmed using umbilical cord DNA and the maternal genotype determined using the TaqMa SNP Genotyping Assay to rs1058587 (Applied Biosystems) according to the manufacturer’s instructions.

### Mass spectrometry studies

Anti-human GDF15 capture antibody (R&D systems, catalogue number: DY957, part no.: 841832) was coupled to tosyl-activated M-280 paramagnetic dynabeads (ThermoFisher Scientific) using the standard supplied protocol. Plasma from each individual (50 µl) was diluted with 150 µl of Buffer E and 5 µl of magnetic beads at 20 mg ml^−1^ was added. Samples were mixed at 850 rpm for 1 h at room temperature on a 96-well MixMate plate mixer (Eppendorf). The beads were concentrated using a magnet and the supernatants removed. The beads were washed twice in 200 µl of buffer E. A final wash with 200 µl of 50 mM ammonium bicarbonate was performed and the supernatant removed. Disulfide bonds were reduced with 75 µl of 10 mM DTT in 50 mM ammonium bicarbonate over a 60 minute incubation at 60 °C, before alkylation with 20 µl of 100 mM iodoacetamide in 50 mM ammonium bicarbonate in the dark for 30 min at room temperature. To digest the polypeptide 10 µl of Glu C enzyme (Worthington) at 100 µg ml^−1^ was added and the samples digested overnight at 37 °C. The digestion was stopped by the addition of 20 µl of 1% formic acid in water.

The plasma samples collected from mothers with homozygous fetuses were analysed on a ThermoFisher Q-Exactive Plus Orbitrap using nanoflow analysis with an Ultimate 3000 LC system. Peptides monitored were ARNGD**H**CPLGPGRCCRLHTVRASLE and ARNGD**D**CPLGPGRCCRLHTVRASLE corresponding to the H-peptide and D-peptide (mutant) respectively. Additionally, a GluC derived peptide was monitored from the murine anti-human GDF15 antibody as a surrogate internal standard with which to generate a peak area ratio for comparing relative peptide levels. This peptide was FKCKVNNKDLPSPIE from the heavy chain. A parallel reaction monitoring method was developed for the GDF15 peptides targeting the [M + 5H]^5+^ ion, which corresponded to 579.08 and 574.67 *m*/*z* for the H- and D-peptides, respectively. The product ions corresponding to the same y18 ion for each peptide (693.6950, 694.0285 and 694.3614 *m*/*z*) were summed for quantitative analysis in the Quan Browser programme (ThermoFisher). The plasma samples from mothers with heterozygous fetuses were analysed on an M-Class LC system (Waters), linked to a Xevo TQ-XS triple quadrupole mass spectrometer (Waters) with an IonKey interface. SRM transitions used for these peptides were 579.24/693.89 and 579.24/747.25 for the H-peptide, 574.82/693.89 and 574.82/623.73 (the first SRM transition for each peptide was used as the quantifier transition) as well as 545.27/682.34 and 545.27/926.37 which targeted the peptide ATHKTSTSPIVKSFNRNEC from the C terminus of the murine antibody κ light chain. In both experiments, peptides from the GDF15 protein were expressed as peak area ratios relative to the murine antibody peptide.

#### Estimating total and maternal derived GDF15 by mass spectrometry

The relative abundance of total GDF15 was determined using the sum of the H- and D-peptides. In studies of homozygote fetuses and heterozygous mothers (at position H202D), the proportion of fetal-derived peptide in the maternal circulation was determined by calculating the proportion of discordant maternal peptide (discordant maternal peptide/total GDF15) and multiplying this by two (to account for the fact that the discordant peptide represents only half of all GDF15 made by the mother).

For pregnancies where the fetus was heterozygous, and the mother was homozygous for the reference allele (HH) at position H202D the proportion of the discordant fetal peptide was calculated by dividing this by total GDF15 and multiplying this by two. Noticing that this produced nonsensical fetal proportions of GDF15 (for example, in excess of 100%) in almost all samples tested, we calculated the average proportion of the D-peptide in each pregnancy and used a one-sample *t*-test to determine whether the D-peptide constituted greater than 50% of total GDF15.

Linear mixed models with random intercepts implemented in the LmerTest package (https://cran.r-project.org/web/packages/lmerTest/index.html) were used to characterize the effect of gestational age on relative abundance of natural-log transformed total circulating GDF15 measured by mass spectrometry.

### Functional studies of C211G

#### Plasmid construction

The expression vector for C-terminally Flag-tagged full-length human GDF15 was obtained from Genscript. The C211G mutant was generated by site-directed mutagenesis of the wild-type vector using the QuikChange II protocol (Agilent). To generate the Myc-tagged versions, the sequences corresponding to Flag tags were replaced by those encoding for Myc tags using the In-Fusion PCR cloning system (Takara) according to the kit’s guidelines. All plasmid sequences were confirmed by direct nucleotide sequencing.

#### Cell culture and transfection

Human embryonic kidney (HEK) 293 T cells were obtained from ECACC/PHE and maintained in DMEM (Gibco) supplemented with 10% fetal bovine serum (Hyclone), 1% penicillin-streptomycin and 2 mM l-glutamine (Invitrogen), in a 5% CO_2_/95% O_2_ atmosphere incubator at 37 °C. All cell lines were routinely tested as negative for mycoplasma contaminations using VenorGem Classic Mycoplasma Testing PCR Kit (Minerva Biolabs).

Cells were transiently transfected using Lipofectamine 3000 (Invitrogen) in 12-well plates with a total of 1,000 ng DNA, as directed by the manufacturer.

#### Immunoblotting and co-immunoprecipitation

At 72 h post-transfection, conditioned media samples were harvested and centrifuged and proteins denatured under reducing conditions at 70 °C. For immunoblotting of intracellular proteins, cells were washed twice with cold PBS and lysed in M-Per Mammalian Protein Extraction Reagent (Thermo Scientific) supplemented with protease inhibitors. Whole cell extracts were sonicated and cleared by centrifugation and protein concentration estimated using the Bio-Rad DC protein assay kit (Bio-Rad Laboratories).

For co-immunoprecipitation experiments, Flag-tagged proteins were immunoprecipitated with anti-Flag magnetic agarose (Pierce Anti-DYKDDDDK Magnetic Agarose, ThermoFisher) according to the manufacturer’s protocol. Elution of bound proteins was performed with reducing SDS–PAGE sample buffer. Proteins were resolved by SDS–PAGE in NuPAGE Novex 4–12% Bis-Tris gels and transferred onto nitrocellulose membranes using the iBlot system (Invitrogen). Membranes were then blocked in 50 mM Tris-HCl, pH 7.6, 150 mM NaCl, 0.1% Tween-20 and 5% non-fat milk for 1 h at room temperature and probed for 18 h at 4 °C with antibodies specific for Flag tag (Sigma-Aldrich catalogue no. F1804, 1:500 dilution), Myc tag (9E10, sc-40, Santa Cruz Biotechnology, 1:1,000 dilution) or calnexin (Cell Signalling Technology catalogue no. 2679, 1:1,000 dilution). Chemiluminescence imaging was conducted using Bio-Rad ChemidDoc XRS+ or MP Imaging systems with Image Lab or Image Lab Touch v.3.0.1 software packages, respectively.

### Mouse studies

In Cambridge, all mouse studies were performed in accordance with UK Home Office Legislation regulated under the Animals (Scientific Procedures) Act 1986 Amendment Regulations 2012 following ethical review by the University of Cambridge Animal Welfare and Ethical Review Body (AWERB).

Adult wild-type C57BL/6 J male or female mice were purchased from Charles River and kept under controlled light (12 h light:dark cycle (06:00:18:00), temperature (22 ± 1 °C) and humidity conditions (45–65%) in individually ventilated cages with ad libitum access to food (RM3(E) Expanded Chow (Special Diets Services)) and water.

On the day of the experiment mice were divided into two weight- and sex-matched groups, single-housed and injected subcutaneously (s.c.) with either vehicle control (PBS) or GDF15 long-acting protein (FC-GDF15) provided by Pfizer Inc. under a material transfer agreement^21^ at the dose of 0.01 mg kg^−1^ (*n* = 17, 12 male, 5 female, in the control; and 19 in the FC_GDF15 group, 13 male, 6 female). Food intake and body weight were measured daily. On the fourth day, human recombinant GDF15 (hrGDF15, catalogue no. Qk017, Qkine) was administered via s.c. injection as a single dose in the afternoon (17:00). In all mice food intake and body weight were measured 16 h after injection of hrGDF15. One cohort of mice (*n* = 7–8 males per group) were killed at 09:00 the morning after the hrGDF15 injection, while the remainder went on to have food intake and body weight measured at 17:00. Human GDF15 was measured using the human GDF15 ELISA (catalogue no. DY957, R&D Systems, Bio-Techne). In the mouse study one female animal assigned to the control group (vehicle) was excluded due to failed subcutaneous injection with human recombinant GDF15. In addition, a food intake data point of another female vehicle control mouse (overnight food intake the day before treatment with human recombinant GDF15) was excluded from the analysis due to a transcription error during data collection.

For the second mouse experiment, C57BL/6N-Gdf15tm1a(KOMP)Wtsi/H mice (*Gdf15*^−/−^ mice) were bred in-house from a line originally obtained from the MRC Harwell Institute. Cohorts of Gdf15^−/−^mice and wild-type littermates on a C57BL6/N background were obtained from het × het breeding pairs. At least 3 days before the start of the experiment male and female animals aged-matched for genotype were single-housed and food intake and body weight monitored. On day 1 of the experiment, all mice received a control injection s.c. at 18:00 and body weight and food intake were measured at 24 h later (day 2). At 18:00 of day 2, all mice received an injection of human recombinant GDF15 (catalogue no. Qk017, Qkine) at a dose of 0.01 mg kg^−1^ s.c., food intake and body weight were measured again at 18:00 the day after. In the GDF15KO mouse study one male homozygous food intake data point was excluded due to a transcription error during data collection and one female homozygous food intake data point was unavailable due to food-handling error (inadvertent disposal of food before weight measurement) during data collection.

Hypothesis testing was conducted using repeated-measures two-way ANOVA or mixed effect models, with post hoc Sidak’s test with the Geisser–Greenhouse correction (where appropriate) implemented in Prism (Graphpad).

### Statistical analyses

Statistical analyses, including software employed, are described in the relevant sections of the text above.

### Reporting summary

Further information on research design is available in the Nature Portfolio Reporting Summary linked to this article.

## Online content

Any methods, additional references, Nature Portfolio reporting summaries, source data, extended data, supplementary information, acknowledgements, peer review information; details of author contributions and competing interests; and statements of data and code availability are available at 10.1038/s41586-023-06921-9.

### Supplementary information

Supplementary Figure Uncropped blots related to Extended Data Fig. 3A and B.

Reporting Summary

Supplementary Tables Supplementary Tables 1–6 and 8–14.

Supplementary Table 7 Demographics of C211G carriers and controls.

### Source data

Source Data Fig. 1

Source Data Fig. 2

Source Data Fig. 3

Source Data Fig. 4

Source Data Extended Data Fig. 2

Source Data Extended Data Fig. 6

## Data Availability

Summary statistics of the GDF15 GWAS in Generation Scotland will be shared in the Generation Scotland DataShare collection (https://datashare.ed.ac.uk/handle/10283/844). For the hyperemesis gravidarum GWAS, qualified researchers can contact apply.research@23andMe.com to gain access to full GWAS summary statistics following an agreement with 23andMe that protects 23andMe participant privacy. The source data files are provided and accompany each figure, except where doing so would result in unauthorized release of summary statistics from the 23andMe HG GWAS. Source data are provided with this paper.

## Code availability

Custom code used for the analyses presented in this manuscript is available at: https://github.com/mancusolab/gdf15_analyses.
