# Supplementary material for: GDF15 linked to maternal risk of nausea and vomiting during pregnancy
Source: Nature. 2023 Dec 13;625(7996):760–7. doi: 10.1038/s41586-023-06921-9 (PMC10808057; doi:10.1038/s41586-023-06921-9)

nature portfolio

https://doi.org/10.1038/s41586-023-06921-9

Supplementary information

GDF15 linked to maternal risk of nausea and vomiting during pregnancy

In the format provided by the authors and unedited

Uncropped blots (related to Extended Data Figures 3A, 3B)

Extended Data Figure 3A:

Calnexin levels are shown in Extended Data Figure 3A as loading controls. Immunoblotting using anti-calnexin antibodies (chemiluminescence) was performed on the portion of the membrane showing molecular weight markers positions larger than ~65 kDa (epi-white illumination).
The red box shows region of interest shown in Extended Data Figure 3A.

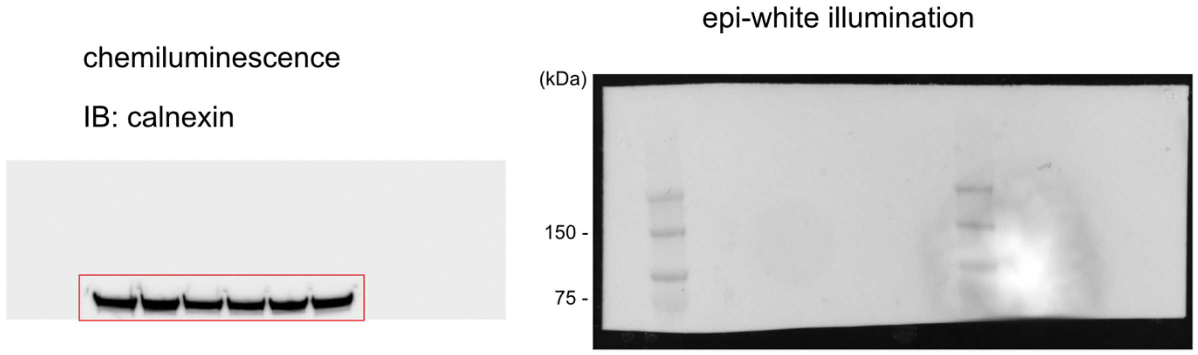

Extended Data Figure 3B:

Calnexin levels are shown in Extended Data Figure 3B as loading controls. Immunoblotting using anti-calnexin antibodies (chemiluminescence) was performed on the portion of the membrane showing molecular weight markers positions larger than ~65 kDa (epi-white illumination).
The red box shows region of interest shown in Extended Data Figure 3B.

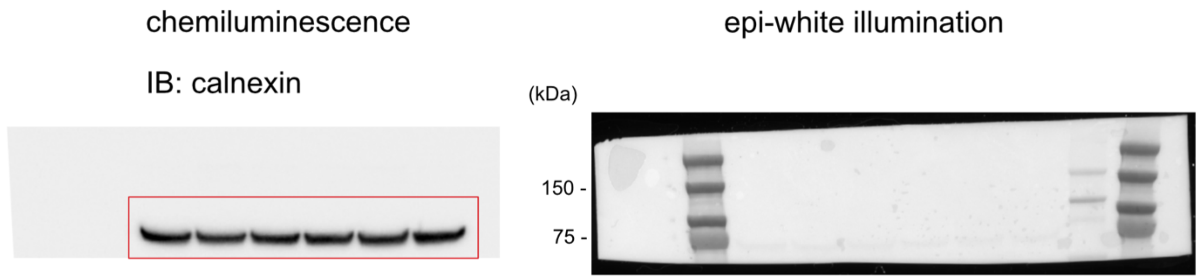

Supplement: Supplementary file 1 — Uncropped blots related to Extended Data Fig. 3A and B. [file 41586_2023_6921_MOESM1_ESM.pdf]
