# Supplementary material for: GDF15 linked to maternal risk of nausea and vomiting during pregnancy
Source: Nature. 2023 Dec 13;625(7996):760–7. doi: 10.1038/s41586-023-06921-9 (PMC10808057; doi:10.1038/s41586-023-06921-9)
Supplement: Supplementary file 2 — Reporting Summary [file 41586_2023_6921_MOESM2_ESM.pdf]

nature portfolio

Corresponding author(s): Professor Stephen O'Rahilly

Last updated by author(s): 12/09/2023

Reporting Summary

Nature Portfolio wishes to improve the reproducibility of the work that we publish. This form provides structure for consistency and transparency in reporting. For further information on Nature Portfolio policies, see our Editorial Policies and the Editorial Policy Checklist.

Statistics

For all statistical analyses, confirm that the following items are present in the figure legend, table legend, main text, or Methods section.

n/a	Confirmed	
☐	☒	The exact sample size (*n*) for each experimental group/condition, given as a discrete number and unit of measurement
☐	☒	A statement on whether measurements were taken from distinct samples or whether the same sample was measured repeatedly
☐	☒	The statistical test(s) used AND whether they are one- or two-sided<br>*Only common tests should be described solely by name; describe more complex techniques in the Methods section.*
☐	☒	A description of all covariates tested
☐	☒	A description of any assumptions or corrections, such as tests of normality and adjustment for multiple comparisons
☐	☒	A full description of the statistical parameters including central tendency (e.g. means) or other basic estimates (e.g. regression coefficient) AND variation (e.g. standard deviation) or associated estimates of uncertainty (e.g. confidence intervals)
☐	☒	For null hypothesis testing, the test statistic (e.g. *F*, *t*, *r*) with confidence intervals, effect sizes, degrees of freedom and *P* value noted<br>*Give P values as exact values whenever suitable.*
☐	☒	For Bayesian analysis, information on the choice of priors and Markov chain Monte Carlo settings
☒	☐	For hierarchical and complex designs, identification of the appropriate level for tests and full reporting of outcomes
☐	☒	Estimates of effect sizes (e.g. Cohen's *d*, Pearson's *r*), indicating how they were calculated

Our web collection on statistics for biologists contains articles on many of the points above.

Software and code

Policy information about availability of computer code

Data collection	Western blot images were acquired using BioRad ChemidDoc XRS+ or MP Imaging systems with Image Lab or Image Lab Touch 3.0.1 software packages, respectively.
Data analysis	The following publicly available software was used for data analysis and visualization: PLINK v1.90b6.26 via the Swiss army knife application in DNANexus' Research Access Platform for UKBB, Stata (version 13.1), Prism v9 (Graphpad), BOLT-LMM-2.2, GIMP-2.10, R v4.2.2 with the following packages: LmerTest v3.1-3, ggplot2 v3.4.0, coloc v5.1.0, tidyverse v3.1.2, DescTools v0.99.47, MendelianRandomization v0.7.0, SusieR v0.12.35.

For manuscripts utilizing custom algorithms or software that are central to the research but not yet described in published literature, software must be made available to editors and reviewers. We strongly encourage code deposition in a community repository (e.g. GitHub). See the Nature Portfolio guidelines for submitting code & software for further information.

Data

Policy information about availability of data

All manuscripts must include a data availability statement. This statement should provide the following information, where applicable:

- Accession codes, unique identifiers, or web links for publicly available datasets
- A description of any restrictions on data availability
- For clinical datasets or third party data, please ensure that the statement adheres to our policy

Summary statistics of the GDF15 GWAS in Generation Scotland will be shared in the Generation Scotland DataShare collection (https://datashare.ed.ac.uk/handle/10283/844). For the hyperemesis gravidarum GWAS: qualified researchers can contact apply.research@23andMe.com to gain access to full GWAS summary statistics following an agreement with 23andMe that protects 23andMe participant privacy, directly relevant replication data are available upon request.

Human research participants

Policy information about studies involving human research participants and Sex and Gender in Research.

Reporting on sex and gender	Biological sex is reported in the human studies. The C211G carriers and controls in the CROATIA-Korcula study were matched for sex. Biological sex was included as a co-variate in the GWAS of GDF15 in generation Scotland.
Population characteristics	Relevant population demographics are described either at a participant level or in an appropriately summarized form in the supplementary tables (Cambridge Baby Growth - Supplementary Table 2, HG Study - Supplementary Table 4, GDF15-MS studies - Supplementary Table 6, C211G carriers and controls in the Croatia-Korcula study - Supplementary Table 7).
Recruitment	Recruitment details are outlined in the methods of the manuscript for new studies reported here. We also used biobanked samples and existing data from existing cohort studies, namely: Cambridge Baby Growth Study, Korcula-CROATIA, Generation Scotland, Pregnancy Outcome and Prevention Study and a Whole-Exome Study of HG and controls. References to the relevant original publications outlining detailed information regarding recruitment have been published and are cited in the body of the manuscript.
Ethics oversight	Ethical approval for the Cambridge Baby Growth Study was granted by the Cambridge Local Research Ethics Committee, Cambridge University Hospitals NHS Foundation Trust, Cambridge, U.K. (00/325). HG Study: Ethical approval was granted by the National Research Ethics Service Committee - East of England, Norfolk, U.K. (14/EE/1247). All procedures followed were in accordance with both institutional and international guidelines. Written informed consent was obtained from all women. CROATIA-Korcula: Ethical approval was given for recruitment of all participants by ethics committees in both Scotland and Croatia. Ethical approval for the Generation Scotland study was obtained from the Tayside Committee on Medical Research Ethics (on behalf of the National Health Service). The prevalence of nausea and vomiting in thalassaemia survery was approved by the Ethics Review Committee of University of Kelaniya, Sri Lanka (Ref: P/228/11/2019). The 23andMe GWAS of HG research participants provided informed consent and volunteered to participate in the research online, under a protocol approved by the external AAHRPP-accredited IRB, Ethical & Independent (E&I) Review Services. As of 2022, E&I Review Services is part of Salus IRB (https://www.versiticlinicaltrials.org/salusirb). All research participants included in the analysis provided informed consent and answered on-line surveys according to a human subjects protocol approved by Ethical & Independent Review Services, a private institutional review board

Note that full information on the approval of the study protocol must also be provided in the manuscript.

Field-specific reporting

Please select the one below that is the best fit for your research. If you are not sure, read the appropriate sections before making your selection.

☒ Life sciences ☐ Behavioural & social sciences ☐ Ecological, evolutionary & environmental sciences

For a reference copy of the document with all sections, see nature.com/documents/nr-reporting-summary-flat.pdf

Life sciences study design

All studies must disclose on these points even when the disclosure is negative.

Sample size	The analysis presented in the Cambridge Baby Growth Study is from a convenience sample of women with completed questionnaires detailing severity of nausea and vomiting in pregnancy and whom provided a blood sample. In the HG study - participants were chosen to maximize the difference in nausea and vomiting scores at a rough ratio of 1:1 cases and controls up to the number of available assay reagents. All available participants carrying a C211G variant who had an available serum sample in the CROATIA-Korcula study (N=10/11) were included in our analysis. We selected 6 age and sex matched controls for each case. For mass spectrometry studies, all pregnancies in the POPS study with discordant foetal/maternal genotypes at H202D were included.

Data exclusions	In the HG study: all participants who had GDF15 measured were included in the primary analysis, comparing GDF15 levels between HG and controls. To study the relationship between gestational age and circulating GDF15 in those with and without HG (Figure 1C), only data on circulating GDF15 from participants studied in the first trimester of pregnancy were included as it was felt that numbers from the second and third trimester of pregnancy were too small to draw reliable inferences.<br><br>In the FC-GDF15 mouse study - one female animal assigned to the control group (vehicle) was excluded due to failed subcutaneous injection with human recombinant GDF15. In addition, a food intake data point of another female vehicle control mouse (overnight food intake the day before treatment with human recombinant GDF15) was excluded from the analysis due to a transcription error during data collection.<br><br>In the GDF15-KO mouse study - one male homozygous food intake data point was excluded due to a transcription error during data collection one female homozygous food intake data point was unavailable due to food-handling error (inadvertent disposal of food before weight measurement) during data collection.
Replication	Cell culture experiments were replicated 3 times. The data presented from mouse studies using FC-GDF15 is from three independent studies (2 male cohorts, 1 female cohort). The data presented from mouse studies using GDF15-KO mice is from four independent studies. In both cases, the trends observed in each independent cohort of mice were directionally consistent with the final result.
Randomization	Age and sex matched mice were allocated to either a Vehicle control or FC-GDF15 group by an investigator at random. For the described cell culture experiments allocation of wells to each plasmid was chosen arbitrarily but was not randomized.
Blinding	For mass spectrometry analyses, researchers were blinded to maternal and foetal genotypes. For studies using GDF15 immunoassays, the researchers were blinded to group allocation/genotype. For the studies using GDF15-KO mice, the investigator measuring food intake and bodyweight was blinded to genotype.

Reporting for specific materials, systems and methods

We require information from authors about some types of materials, experimental systems and methods used in many studies. Here, indicate whether each material, system or method listed is relevant to your study. If you are not sure if a list item applies to your research, read the appropriate section before selecting a response.

Materials & experimental systems

n/a	Involved in the study	
☐	☒	Antibodies
☐	☒	Eukaryotic cell lines
☒	☐	Palaeontology and archaeology
☐	☒	Animals and other organisms
☒	☐	Clinical data
☒	☐	Dual use research of concern

Methods

n/a	Involved in the study	
☒	☐	ChIP-seq
☒	☐	Flow cytometry
☒	☐	MRI-based neuroimaging

Antibodies

Antibodies used	Monoclonal ANTI-Flag clone M2 antibody produced in mouse from Sigma-Aldrich, with catalog code F1804. Mouse monoclonal ANTI-Myc antibody (9E10) with catalog code sc-40 from Santa Cruz Biotechnology. The murine anti-human GDF15 antibody used in mass spectrometry experiments is the capture antibody from an R&D GDF15 ELISA (cat no: DY957). Calnexin antibody: Cell Signal Technology Calnexin (C5C9) Rabbit mAb #2679.
Validation	Validation data for ANTI-Flag clone M2 antibody can be found at https://www.sigmaaldrich.com/GB/en/product/sigma/f1804. Validation data for ANTI-Myc (9E10) mouse monoclonal antibody can be found at https://www.scbt.com/p/c-myc-antibody-9e10. Validation data for calnexin antibody is available at: https://www.cellsignal.com/products/primary-antibodies/calnexin-c5c9-rabbit-mab/2679. The murine anti-human GDF15 antibody was used to pull down GDF15 from plasma which was then detected by mass spectrometry. LC/MS traces of these experiments are available in Figure 2, Supplementary Figure 1. Validation of equivalent recognition of GDF15 H and D peptide using this approach is displayed in Supplementary Figure 2C.

Eukaryotic cell lines

Policy information about cell lines and Sex and Gender in Research

Cell line source(s)	Human embryonic kidney (HEK) 293T cells were obtained from European Collection of Authenticated Cell Cultures (ECACC)/ Public Health England (PHE) with reference for citation in publications: 293T (ECACC 12022001)
Authentication	Cells were obtained from the authenticated general collection of cells of the European Collection of Authenticated Cell Cultures (ECACC). Authentication was carried out by ECACC's AuthentiCell authentication service by morphology and STR-PCR-based profiling (Amelogenin, CSF1PO, D13S317, D16S539, D5S818, D7S820, THO1, TPOX, vWA)
Mycoplasma contamination	Cells tested negative for mycoplasma contaminations using the PCR-based VenorGeM kit.

Commonly misidentified lines (See ICLAC register)	No commonly misidentified cell lines were used in this study.

Animals and other research organisms

Policy information about studies involving animals; ARRIVE guidelines recommended for reporting animal research, and Sex and Gender in Research

Laboratory animals	We used adult male and female C57BL/6 mice aged 10-33 weeks old. Mice were matched according to age and sex for allocation to treatment groups as described above and in the methods.
Wild animals	This study did not use wild animals
Reporting on sex	Both male and female animals were used in this study - the numbers of each are indicated in the methods and figure legends.
Field-collected samples	This work did not involve field collected samples.
Ethics oversight	All mouse studies were performed in accordance with UK Home Office Legislation regulated under the Animals (Scientific Procedures) Act 1986 Amendment Regulations 2012 following ethical review by the University of Cambridge Animal Welfare and Ethical Review Body (AWERB).

Note that full information on the approval of the study protocol must also be provided in the manuscript.
