# Supplementary material for: GDF15 linked to maternal risk of nausea and vomiting during pregnancy
Source: Nature. 2023 Dec 13;625(7996):760–7. doi: 10.1038/s41586-023-06921-9 (PMC10808057; doi:10.1038/s41586-023-06921-9)
Supplement: Supplementary file 3 — Supplementary Tables 1–6 and 8–14. [file 41586_2023_6921_MOESM3_ESM.pdf]

Supplementary Table 1 Recovery of synthetic GDF15 peptides by the MSD R&D DuoSet ELISA and the Ansh Total GDF15 ELISA.

Peptide	Theoretical pg/ml	MSD R&D DuoSet GDF15 Analysed in 2021			Ansh Total GDF15 Analysed in September 2022		
		Measured	Recovery to theoretical	Recovery to HH	Measured	Recovery to theoretical	Recovery to HH
		pg/ml	%	%	pg/ml	%	%
HH	2000	3329	166	NA	2270	114	NA
HH	667	1027	154	NA	677	101	NA
HH	222	367	165	NA	216	97	NA
HH	74	121	164	NA	81	109	NA
HD	2000	2384	119	72	2768	138	122
HD	667	795	119	77	829	124	124
HD	222	282	127	77	266	120	123
HD	74	88	119	73	90	121	111
DD	2000	1269	63	38	2009	100	89
DD	667	446	67	43	573	86	85
DD	222	155	70	42	164	74	76
DD	74	55	74	45	57	76	70

HH: Homodimeric peptide containing the reference H amino acid at position 6 of mature GDF15 (position 202 of the unprocessed form). HD: This is an admixture of synthetic peptides in the approximate ratio of HH:HD:DD of 1:2:1, akin to what would be expected in the circulation of heterozygote carriers of the H202D variant if all possible conformations of the peptide are secreted and eliminated equally. DD: Homodimeric peptide containing the alternate D amino acid at position 6 of mature GDF15. Recovery is expressed to the nearest %. The performance of the R&D DuoSet® assay adapted for use on the Meso Scale Discovery® (MSD) platform is presented for comparison [11].

Supplementary Table 2 Characteristics of participants in the Cambridge Baby Growth Study

Characteristic	Reported No Nausea or Vomiting in Pregnancy (n=148)	Reported Vomiting in Pregnancy (n=168)	P-value
Age (years)	34.0 (33.2-34.6)	32.9 (32.2-33.5)	0.04
Parity (n 0/1/2/>2)	82/52/12/2	75/73/14/6	0.3
Gestational age when GDF15 measured (weeks)	14.9 (14.6-15.1)	15.0 (14.8-15.2)	0.4
Pre-pregnancy BMI (kg/m^2)	23.8 (23.1-24.6)	24.1 (23.5-24.8)	0.5
Reported taking anti-emetics in pregnancy (n yes/no)	0/148	18/150	<0.001
Reported smoking in pregnancy (n yes/no)	5/143	9/159	0.4

Data are mean (95% confidence interval) or numbers. P-values are derived from linear regression models for continuous variables. Rates of anti-emetic usage and smoking status were compared between groups using Fisher's exact and Chi-squared test, respectively.

Supplementary Table 3 Serum GDF15 concentrations in women in the Cambridge Baby Growth study

Statistical Model	Serum GDF15 Concentrations (pg/mL)		P-value
	Women with no nausea or vomiting (n=148)	Women with vomiting (n=168)	
Unadjusted	13,198 (12,421-14,022)	15,442 (14,588- 16,346)	$2.4x10^{-4}$
Adjusted for gestational age	13,181 (12,395-14,018)	15,431 (14,573-16,339)	$2.5x10^{-4}$
Adjusted for gestational age and BMI	12,961 (12,161-13,813)	15,542 (14,663-16,474)	$4.3x10^{-5}$

Data are geometric means (95% confidence intervals). P-values and adjusted GDF15 concentrations are derived from linear regression models of natural-log transformed GDF15.

Supplementary Table 4

Characteristics of cases and controls from the Hyperemesis Gravidarum study

Characteristic	Reported Minimal Nausea or Vomiting in Pregnancy (n=56)	Hyperemesis Gravidarum (n=57)	P-value
Age (years)	31.6 (30.1-33.1)	29.7 (28.2-31.2)	0.09
Pregnancy length when GDF15 measured (weeks)	10.1 (8.9-11.4)	9.0 (7.8-10.2)	0.2
Reported taking anti-emetics in current pregnancy (n yes/no)	0/56	38/19	<0.001
Reported requiring rehydration in current pregnancy (n yes/no)	1/55	23/34	<0.001
"Current Nausea" Score at time of sampling (out of 10)	0.2 (0-0.7)	6.0 (5.5-6.5)	$3.6x10^{-31}$
"Worst Nausea" in pregnancy Score (out of 10)	1.6 (1.2-2.0)	9.4 (9.0-9.8)	$5.0x10^{-51}$
"Current Vomiting" Score at time of sampling (out of 10)	0 (0-0.7)	3.5 (2.9-4.2)	$2.1x10^{-11}$
"Worst Vomiting" in pregnancy Score (out of 10)	0.1 (0-0.5)	8.7 (8.3-9.1)	$1.0x10^{-54}$
Sum of Nausea and Vomiting Scores (out of 40)	1.9 (0.7-3.0)	27.6 (26.5-28.7)	$1.1x10^{-57}$
Reported smoking in pregnancy (n yes/no)	3/53	11/46	0.04

Data are mean (95% confidence interval) or numbers. P-values are from linear regression for continuous variables and Fisher's exact test for categorical variables.

Supplementary Table 5

Serum GDF15 concentrations in women in the hyperemesis gravidarum study

Statistical Model	Serum GDF15 Concentrations (pg/mL)		P-value
	Women with minimal nausea or vomiting (n=56)	Women with HG (n=57)	
Unadjusted	9,396 (7,981-11,062)	13,172 (11,204-15485)	$4.3x10^{-3}$
Adjusted for gestational age	8,899 (7,674-10,320)	13,314 (11,512-15,399)	$2.1x10^{-4}$
Adjusted for gestational age and smoking status	8,630 (7,448-9,999)	13,595 (11,778-15,692)	$3.3x10^{-5}$

Data are geometric mean (95% confidence interval). P-values and adjusted GDF15 concentrations are derived from linear regression models of natural-log transformed GDF15.

Supplementary Table 6

Genotyping results obtained using placental RNAseq data to select samples used for MS-based measurement of GDF15 in maternal plasma during pregnancy. Fetal genotypes were confirmed by PCR using umbilical cord DNA as described in the methods.

Maternal age at enrolment	Ref.allele	Alt.allele	RefCount	AltCount	Fetal Genotype	Maternal Genotype
32	C	G	42	49	C/G	C/C
22	C	G	55	63	C/G	C/C
30	C	G	71	46	C/G	C/C
24	C	G	65	56	C/G	C/C
31	C	G	63	57	C/G	C/C
32	C	G	64	51	C/G	C/C
26	C	G	65	58	C/G	C/C
24	C	G	36	46	C/G	C/C
28	C	G	41	44	C/G	C/C
27	C	G	41	64	C/G	C/C
29	C	G	47	41	C/G	C/C
37	C	G	68	58	C/G	C/C
39	C	G	0	118	G/G	C/G
31	C	G	1	101	G/G	C/G
29	C	G	0	100	G/G	C/G
36	C	G	2	85	G/G	C/G
25	C	G	2	29	G/G	C/G
20	C	G	1	26	G/G	G/G
32	C	G	129	0	C/C	C/C
34	C	G	126	0	C/C	C/C
30	C	G	125	0	C/C	C/C
29	C	G	125	0	C/C	C/C
28	C	G	124	0	C/C	C/C
25	C	G	121	0	C/C	C/C
31	C	G	116	0	C/C	G/C
25	C	G	110	0	C/C	G/C

Supplementary Table 8. The effect of fetal genotype on nausea and vomiting in pregnancy in mothers carrying the C211G variant in *GDF15*.

Mother TG (C211G)	Child	Child Genotype	Mother HG	Prescription Antiemetic	IV Fluids	Emergency Room	Hospitalized	Symptom Resolution
1	1.1	TT	Y	Y	Y	Y	Y	at birth
	1.2	TT	Y	Y	Y	Y	Y	at birth
2	2.1	TG	N	N	N	N	N	T1
	2.2	TT	Y	Y	Y	Y	Y	T3
3	3.1	TG	Y	Y	Y	Y	N	T2
	3.2	TT	Y	Y	Y	Y	N	T3
	3.3	TG	Y	Y	Y	Y	N	after birth
	3.4	TT	Y	Y	N	N	N	after birth
	3.5	TT	Y	Y	Y	Y	N	after birth
4	4.1	TG	Y	Y	Y	N	N	after birth
	4.2	TG	Y	Y	Y	N	N	after birth
	4.3	TT	Y	Y	Y	N	Y	after birth
	4.4	TT	Y	Y	N	N	N	after birth
5	5.1	TT	Y	Y	Y	Y	N	T2
	5.2	TT	Y	Y	Y	Y	Y	T2
	5.3	TG	N	N	N	N	N	T1
6	6.1	TG	N	N	N	N	N	T1

C211G (TG) Mothers have Hyperemesis Gravidarum with all 10 pregnancies carrying a homozygous TT fetus, but normal NVP (no treatment) in 3/7 pregnancies carrying a TG fetus. (T1,2,3=Trimester 1, 2, or 3). IV fluids – Intravenous Fluids.

Supplementary Table 9 Mendelian Randomization of circulating GDF15 levels on hyperemesis gravidarum risk using the Roche assay

Method	Slope/Intercept	Estimate	Std Error	95% CI		P-value
Simple median	slope	-0.79	0.05	-0.88	-0.70	8.47E-66
Weighted median	slope	-0.95	0.04	-1.03	-0.87	1.55E-113
Penalized weighted median	slope	-0.95	0.04	-1.03	-0.87	1.56E-113
IVW	slope	-0.35	0.04	-0.43	-0.27	6.98E-17
Penalized IVW	slope	-0.35	0.04	-0.43	-0.27	6.98E-17
Robust IVW	slope	-0.35	0.04	-0.43	-0.27	6.98E-17
Penalized robust IVW	slope	-0.35	0.04	-0.43	-0.27	6.98E-17
MR-Egger	slope	-0.33	0.04	-0.41	-0.25	2.00E-15
MR-Egger	intercept	0.00	0.00	-0.01	0.00	2.94E-02
Penalized MR-Egger	slope	-0.33	0.04	-0.41	-0.25	2.00E-15
Penalized MR-Egger	intercept	0.00	0.00	-0.01	0.00	2.94E-02
Robust MR-Egger	slope	-0.33	0.04	-0.41	-0.25	2.00E-15
Robust MR-Egger	intercept	0.00	0.00	-0.01	0.00	2.94E-02
Penalized robust MR-Egger	slope	-0.33	0.04	-0.41	-0.25	2.00E-15
Penalized robust MR-Egger	intercept	0.00	0.00	-0.01	0.00	2.94E-02

We estimated putative causal effects (ie slope) of circulating GDF15 levels in the non-pregnant state on hyperemesis gravidarum (HG) risk using m=259 variants from Roche-based pQTL summary data (n=18,184) and 23andMe HG summary data (n=17,062). Harmonized pQTL, GWAS, and LD estimated from UK Biobank WGS individuals. The causal effect estimates represent the change in HG risk in log-odds per standard deviation increase in circulating GDF15.

Supplementary Table 10 Mendelian Randomization of circulating GDF15 levels on hyperemesis gravidarum risk are robust to LD reference

Method	LD Reference	Estimate	Std Error	95% CI		P-value
IVW	UKBB	-0.35	0.04	-0.43	-0.27	6.98E-17
IVW	1000G	-0.36	0.03	-0.42	-0.30	9.41E-30

To assess the stability of our results to choice of LD reference panel, we re-estimated putative causal effects (ie slope) of circulating GDF15 levels in the non-pregnant state on hyperemesis gravidarum (HG) risk using Roche-based pQTL summary data (n=18,184) and 23andMe HG summary data (n=17,062). However, LD was estimated from 1000G WGS individuals which resulted in m=310 variants (see Methods). The causal effect estimates represent the change in HG risk in log-odds per standard deviation increase in circulating GDF15.

Supplementary Table 11 IVW MR results using LD-Aware MR and LD-Aware MR focusing on SNPs within credible sets

Method	Estimate	Std Error	95% CI		P-value	Conditional SNP	SNP instruments
IVW	-0.35	0.04	-0.43	-0.27	6.98E-17	-	Standard
IVW	-1.00	0.12	-1.23	-0.76	5.58E-17	-	SuSiE credible set
IVW	-0.21	0.02	-0.25	-0.16	3.82E-19	rs1058587	Standard
IVW	-0.12	0.03	-0.18	-0.06	1.94E-04	rs45543339	Standard
IVW	-1.13	0.01	-1.15	-1.11	P<2E-18	rs1058587	SuSiE credible set
IVW	-1.12	0.01	-1.14	-1.11	P<2E-18	rs45543339	SuSiE credible set

Estimate refers to the causal estimate from IVW MR. Conditional SNP refers to GWAS/pQTL results after residualizing out effect from reported SNP. SNP instruments refers to using the p-value threshold (i.e. "Standard") compared with SNPs found in SuSiE credible sets.

Supplemental Table 12 Mendelian Randomization of circulating GDF15 levels on hyperemesis gravidarum risk are robust to the protein altering variant p.H202D (rs1058587)

Method	Analysis	Estimate	Std Error	95% CI		P-value
IVW	Marginal	-0.35	0.04	-0.43	-0.27	6.98E-17
IVW	Conditional	-0.21	0.02	-0.25	-0.16	3.82E-19

To assess the stability of our results to variant rs1058587, which was previously suggested to confound quantification [40, 41], we re-estimated putative causal effects (ie slope) of circulating GDF15 levels in the non-pregnant state on hyperemesis gravidarum (HG) risk using m=258 variants from Roche-based pQTL summary data (n=18,184) and 23andMe HG summary data (n=17,062) after conditioning (ie residualizing) on variant rs1058587 (see Methods). Results from IVW on reported pQTL/GWAS data are listed under "Marginal", while updated MR results obtained from a conditional analysis are listed under "Conditional". The causal effect estimates represent the change in HG risk in log-odds per standard deviation increase in circulating GDF15.

Supplementary Table 13 Colocalization analysis identifies two shared genetic signals for circulating GDF15 and hyperemesis gravidarum risk

hit.pQTL	hit.GWAS	PP.H0.abf	PP.H1.abf	PP.H2.abf	PP.H3.abf	PP.H4.abf
rs16982345	rs45543339	7.14E-231	1.72E-14	2.75E-219	4.63E-03	9.95E-01
rs1227734	rs1227731	1.07E-189	3.75E-04	2.09E-188	5.31E-03	9.94E-01

Multi-SNP colocalization analyses was performed using the R package coloc (see Methods). PP.HX.abf corresponds to the posterior probability that a variant cluster is null (X=0), is private to GDF15 levels (X=1), hyperemesis gravidarum (HG) risk (X=2), contributes independently to GDF15 levels and HG risk (X=3), and colocalizes across both traits (X=4), with a posterior probability of >0.8 generally considered as strong evidence of colocalization.

Supplementary Table 14

The prevalence of nausea, vomiting and loss of appetite of females with thalassaemia

Parameter	Thalassaemia group (n=20)	Non-thalassaemia group (n=20)	Adjusted odds ratio (95% CI)	P-value
Nausea	1 (5%)	12 (60%)	0.026 (0.002-0.310)	0.004#
Vomiting	1 (5%)	13 (65%)	0.021 (0.002-0.248)	0.002#
Loss of appetite	3 (15%)	13 (65%)	0.066 (0.010-0.443)	0.005#
Nausea and vomiting persistent beyond the first trimester	1 (5%)	3 (15%)	0.345 (0.024-4.987)	0.435#
Nausea and vomiting persistent throughout pregnancy	0	1 (5%)	-	1.00*
Nausea and vomiting requiring treatment	0	6 (30%)	-	0.020*
Nausea and vomiting requiring hospitalisation	0	3 (15%)	-	0.231*

Non-thalassaemia group: control group without thalassemia, matched for age and ethnicity. The prevalence of symptoms is adjusted for parity, number of children and time since index pregnancy. # Logistic regression * Fisher's exact test
